# Supplementary material for: Co-existing mental and somatic conditions in Swedish children with the avoidant restrictive food intake disorder phenotype
Source: medRxiv. 2024 Mar 15:2024.03.10.24304003. Preprint. [Version 1] doi: 10.1101/2024.03.10.24304003 (PMC10980122; doi:10.1101/2024.03.10.24304003)
Supplement: Supplement 1 [file media-1.pdf]

Supplementary Material to *"Co-existing mental and somatic conditions in Swedish children and adolescents with the avoidant restrictive food intake disorder phenotype"*

Authors

Marie-Louis Wronski, MSc[a,b,c]; Ralf Kuja-Halkola, PhD[a]; Elin Hedlund, BA[a]; Miriam I. Martini, MSc[a]; Paul Lichtenstein, PhD[a]; Sebastian Lundström, PhD[d]; Henrik Larsson, PhD[a,e]; Mark J. Taylor, PhD[a], Nadia Micali, MD, PhD[f,g,h]; Cynthia M. Bulik, PhD[a,i,j]; Lisa Dinkler, PhD[a,]*****

[a] Department of Medical Epidemiology and Biostatistics, Karolinska Institutet, Stockholm, Sweden

[b] Neuroendocrine Unit, Department of Medicine, Massachusetts General Hospital and Harvard Medical School, Boston, MA, USA

[c] Translational Developmental Neuroscience Section, Division of Psychological and Social Medicine and Developmental Neurosciences, Faculty of Medicine, TU Dresden, Dresden, Germany

[d] Gillberg Neuropsychiatry Centre, Institute of Neuroscience and Physiology, Sahlgrenska Academy, University of Gothenburg, Gothenburg, Sweden

[e] School of Medical Sciences, Örebro University, Örebro, Sweden

[f] Center for Eating and Feeding Disorders Research, Mental Health Center Ballerup, Copenhagen University Hospital – Mental Health Services CPH, Denmark

[g] Institute for Biological Psychiatry, Mental Health Centre Sct Hans, Copenhagen University Hospital – Mental Health Services, Copenhagen, Denmark

[h] Great Ormond Street Institute of Child Health, University College London, London, United Kingdom

[i] Department of Psychiatry, University of North Carolina at Chapel Hill, NC, USA

[j] Department of Nutrition, University of North Carolina at Chapel Hill, NC, USA

***** Corresponding author: Lisa Dinkler, PhD, Department of Medical Epidemiology and Biostatistics, Karolinska Institutet, Nobels Väg 12A, 17177 Stockholm, Sweden (lisa.dinkler@ki.se)

Table of contents

Supplementary element	Content	Page
Table S1	Descriptive statistics of the sample	3
Table S2	Diagnostic classification of examined mental and somatic conditions in ARFID and controls	4
Table S3	Prevalence of mental and somatic conditions in ARFID and controls overall, per sex, and per age group	11
Table S4	Base Cox regression model estimates in ARFID vs. controls	14
Table S5	Cumulative incidences of mental and somatic conditions before ages 6, 12, and 18 years in ARFID and controls	16
Table S6	Number of distinct overall, all mental, and all somatic diagnoses in ARFID vs. controls	19
Table S7	Number of inpatient days due to any, any mental, and any somatic diagnosis in ARFID vs. controls	20
Table S8	Sex-stratified Cox regression model estimates in ARFID vs. controls	21
Table S9	Sex effects on number of distinct overall, mental, and somatic diagnoses in ARFID vs. controls	23
Table S10	Sex effects on number of inpatient days due to any, any mental, and any somatic diagnosis in ARFID vs. controls	24
Table S11	Age group-stratified Cox regression model estimates in ARFID vs. controls	25
Figure S1	Cumulative incidence plots in ARFID vs. controls for individual and grouped conditions	26
Figure S2	Age-varying hazard ratios in ARFID vs. controls for ICD-chapter F: mental conditions	28

Definition of terms used in the Manuscript and Supplementary Material

ARFID, children and adolescents *with* the avoidant restrictive food intake disorder phenotype; controls, children and adolescents *without* the avoidant restrictive food intake disorder phenotype; sex, sex assigned at birth.

Short labels for outcomes

Collapsed labels to indicate analyzed conditions in a space-efficient manner (applicable to all figures and tables in the Manuscript and Supplementary Material):

ChromosomalAbnorm, chromosomal abnormalities; Malform, malformation; NoninfectiveIBD, noninfective inflammatory bowel disease; ObsessCompulsDisorder, obsessive-compulsive disorder; OtherUpperGastrointest, other upper gastrointestinal conditions; Perinat, perinatal; PerinatCNSConditions, perinatal conditions of the central nervous system; PerinatRespiratCardiovasc, perinatal respiratory and cardiovascular conditions; ScholastDevelopment, scholastic development; VascArterial, vascular disorders of the arterial system; VascVenous, vascular disorders of the venous system.

Table S1. Descriptive statistics of the sample

**Characteristic** n (%)	**1. Overall**	**2. ARFID**	**Controls**	**3. ARFID**		**4. Controls**		**Test statistics**		
		**Overall**	**Overall**	**Female**	**Male**	**Female**	**Male**	**Test**	χ^2	**p-value**
A. Full sample	30,795 (100.00%)	616 (2.00%)	30,179 (98.00%)	241 (39.12%)	375 (60.88%)	14,922 (49.44%)	15,257 (50.56%)	Difference in sex distribution in ARFID vs. controls (overall)	25.32	<0.0001
B. Age at CATSS assessment										
- 9 years	24,314 (100.00%)	512 (2.11%)	23,802 (97.89%)	203 (39.65%)	309 (60.35%)	11,816 (49.64%)	11,986 (50.36%)	Difference in CATSS assessment age in ARFID vs. controls (overall)	6.30	0.012
- 12 years	6,481 (100.00%)	104 (1.60%)	6,377 (98.40%)	38 (36.54%)	66 (63.46%)	3,106 (48.71%)	3,271 (51.29%)			
C. Birth year										
- 1992-1996	9,469 (100.00%)	154 (1.63%)	9,315 (98.37%)	54 (35.06%)	100 (64.94%)	4,534 (48.67%)	4,781 (51.33%)	Difference in birth year distribution in ARFID vs. controls (overall)	14.27	0.003
- 1997-2000	7,739 (100.00%)	149 (1.93%)	7,590 (98.07%)	71 (47.65%)	78 (52.35%)	3,759 (49.53%)	3,831 (50.47%)			
- 2001-2004	7,327 (100.00%)	161 (2.20%)	7,166 (97.80%)	60 (37.27%)	101 (62.73%)	3,561 (49.69%)	3,605 (50.31%)			
- 2005-2008	6,260 (100.00%)	152 (2.43%)	6,108 (97.57%)	56 (36.84%)	96 (63.16%)	3,068 (50.23%)	3,040 (49.77%)			

Absolute number (n) and percentage of individuals with ARFID and controls are stated overall and per sex, age at CATSS assessment, and birth year. Row-wise percentages were calculated (separately for 1., 2., 3., and 4.). Birth years were pooled in groups of five (first group 1992–1996) or four (all other groups) consecutive years. Pearson χ^2 tests were conducted to compare counts (highlighted with light-gray background) in ARFID vs. controls. *Abbreviations: ARFID, avoidant restrictive food intake disorder; CATSS, Child and Adolescent Twin Study in Sweden*.

Table S2. Diagnostic classification of examined mental and somatic conditions in ARFID and controls

ICD-chapters					Individual and grouped conditions within ICD-chapters						Comments
**Label**	**Description**	**ICD-10-codes**	**ICD-9-codes**	**Included as outcome?**	**Short label**	**Description**	**ICD-10-codes**	**ICD-9-codes**	n_{ARFID}	**Included as outcome?**	
**Cancer**	Malignant Neoplasms	C00-C97, D00-D09	140-209, 230-234	Yes (malignant neoplasms and in situ neoplasms)	n/a	n/a	n/a	n/a	13	n/a	Chapter-level analysis; individual diagnoses were not analyzed (low prevalence)
**Endocrine Conditions**	Endocrine, nutritional, and metabolic conditions	E00-E90	240-279	Yes	Cystic Fibrosis	Cystic fibrosis	E84	277.0	<5	No (low prevalence)	
					**Diabetes Mellitus**	Diabetes mellitus	E10, E11, E12, E13, E14	250	5	Yes	
					**Lactose Intolerance**	Lactose intolerance	E73	271.3	11	Yes	
					Lipid Metabolism	Disorders of lipoprotein and fatty acid metabolism	E71.3, E78	272	<5	No (low prevalence)	
					**Obesity**	Obesity	E66	278	7	Yes	
					OtherFluid Homeostasis	Disturbances of fluid, electrolyte, acid-base balance (excl. volume depletion)	E87	276, excl. 276.5	<5	No (low prevalence)	
					**Pituitary Conditions**	Conditions of the pituitary gland	E23	253	9	Yes	
					**Puberty Onset**	Disorders of puberty (delayed/precocious)	E30	259.0, 259.1	11	Yes	
					**ShortStature**	Short stature	E34.3	259.4	28	Yes	
					**Thyroid Conditions**	Thyroid conditions	E00, E01, E02, E03, E04, E05, E06, E07	240, 241, 242, 243, 244, 245, 246	7	Yes	
					**Volume Depletion**	Volume depletion (abnormally decreased volume of circulating fluid [plasma])	E86	276.5	24	Yes	
**Mental Conditions**	Mental and behavioral conditions	F30-F39, F40-F42, F45, F50, F70-F79, F80-F89, F90-F98	296-299, 300, 307, 311-315, 317-319	Yes (excl.: F00-F29 = organic mental disorders, psychoactive substance use, schizo-phrenia; F60 = adult personality and behavior)	**ADHD**	Attention deficit hyperactivity disorder	F90	314	108	Yes	
					**Autism**	Autism (incl. childhood autism, atypical autism, Asperger syndrome, other pervasive developmental disorders)	F84.0, F84.1, F84.5, F84.8, F84.9	299.0	85	Yes	
					Bipolar Disorder	Bipolar disorder	F30, F31	296	<5	No (low prevalence)	
					Bulimia Nervosa	Bulimia nervosa	F50.2, F50.3	n/a	<5	No (low prevalence)	Bulimia nervosa diagnosis according to ICD-9 (307.51) could not be ordered from Swedish Twin Registry (greatest level of detail

											provided was 307.5 [4 digits] = other and unspecified disorders of eating)
					**Conduct Disorder**	Conduct disorder	F91	312	9	Yes	
					**Depression**	Depressive episode (single/recurrent), dysthymia	F32, F33, F34.1	296.2, 296.3, 300.4	16	Yes	
					**Enuresis Encopresis**	Enuresis (nonorganic)	F98.0	307.6	17	Yes	Related conditions, combined due to low prevalence
						Encopresis (nonorganic)	F98.1	307.7			
					**Intellectual Disability**	Intellectual disability	F70, F71, F72, F73, F74, F75, F76, F77, F78, F79	317, 318, 319	56	Yes	
					**Motor Development**	Developmental disorders of motor function	F82	315.4	18	Yes	
					**ObsessCompuls Disorder**	Obsessive-compulsive disorder	F42	300.3	5	Yes	
					**OtherAnxiety Conditions**	Anxiety disorders excl. phobias (e.g., panic disorder, generalized anxiety disorder)	F41	300.0	20	Yes	
					**OtherBehavior Emotion**	Other and unspecified behavioral and emotional disorders with onset in childhood and adolescence (e.g., Pica, impaired emotional/social functioning)	F93, F94, F98.3, F98.8, F98.9	313	14	Yes	
					**Other Development**	Mixed specific, other, and unspecified developmental disorders (e.g., developmental agnosia)	F83, F88, F89	315.5, 315.8, 315.9	15	Yes	
					**Phobias**	Phobic anxiety disorders (e.g., social phobia)	F40	300.2	5	Yes	
					**Scholast Development**	Developmental disorders of scholastic skills (reading, spelling, arithmetical)	F81	315.0, 315.1, 315.2	13	Yes	
					Somatoform Disorders	Somatoform disorders	F45	300.8	<5	No (low prevalence)	
					**Speech Development**	Developmental disorders of speech or language (incl. cluttering and stuttering)	F80, F98.5, F98.6	315.3	25	Yes	
					**TicDisorders**	Tic disorders incl. Tourette syndrome	F95	307.2	14	Yes	
Neurologic Conditions	Conditions of the nervous system	See condition-level for specification	See condition-level for specification	No (only selected conditions examined)	**CerebralPalsy**	Cerebral palsy and paralytic syndromes	G80, G81, G82, G83	342, 343, 344	25	Yes	
					**Epilepsy**	Epilepsy	G40	345	36	Yes	
**Circulatory Conditions**	Conditions of the circulatory system	I00-I99	390-459	Yes	**Heart Pulmonary**	Heart and pulmonary heart conditions	I26, I27, I28, I30, I31, I32, I33, I34, I35, I36, I37, I38, I39, I40, I41, I42,	415, 416, 417, 420, 421, 422, 423, 424, 425,	12	Yes	

							I43, I44, I45, I46, I47, I48, I49, I50, I51, I52	426, 427, 428, 429			
					**VascArterial**	Cerebrovascular disorders, disorders of arteries/arterioles/capillaries, hypertension	I10, I11, I12, I13, I14, I15, I60, I61, I62, I63, I64, I65, I66, I67, I68, I69, I70, I71, I72, I73, I74, I75, I76, I77, I78, I79	401, 402, 403, 404, 405, 430, 431, 432, 433, 434, 435, 436, 437, 438, 440, 441, 442, 443, 444, 445, 446, 447, 448, 449	6	Yes	
					**VascVenous Lymphatic**	Disorders of veins, lymphatic vessels, and lymph nodes	I80, I81, I82, I83, I84, I85, I86, I87, I88, I89	451, 452, 453, 454, 455, 456, 457, 458, 459	7	Yes	
**Respiratory Conditions**	Conditions of the respiratory system	J00-J99	460-519	Yes	**AcuteLower Respiratory**	Acute lower respiratory disorders (e.g., pneumonia, influenza, bronchitis)	J09, J10, J11, J12, J13, J14, J15, J16, J17, J18, J20, J21, J22	466, 480, 481, 482, 483, 484, 485, 486, 487, 488, 490	112	Yes	
					**AcuteUpper Respiratory**	Acute upper respiratory infections (e.g., nasopharyngitis, non-streptococcal tonsillitis, laryngitis, and epiglottitis)	J00, J01, J02, J03, J04, J05, J06, excl. J03.0	460, 461, 462, 463, 464, 465	187	Yes	Related conditions, combined for context
						Streptococcal tonsillitis	J03.0, B95.0	034.0			
					**Asthma**	Asthma	J45, J46	493	128	Yes	
					**ChronicLower Respiratory**	Chronic lower respiratory disorders (e.g., chronic bronchitis, chronic obstructive pulmonary disorder)	J40, J41, J42, J43, J44, J47	491, 492, 494, 496	8	Yes	
					**ChronicUpper Respiratory**	Chronic upper respiratory disorders (e.g., chronic sinusitis, chronic rhinitis, chronic nasopharyngitis, polyp, deviated nasal septum, disorders of vocal cords/larynx)	J31, J32, J33, J34, J37, J38, J39	470, 471, 472, 473, 476, 478	94	Yes	Related conditions, combined due to low prevalence
						Chronic disorders of tonsils and/or adenoids (e.g., inflammation, hypertrophy, peritonsillar abscess)	J35, J36	474, 475			
					OtherRespiratory Conditions	Other respiratory conditions (e.g., affecting interstitial tissue and pleura, pulmonary edema, lung abscess, pyothorax, pneumothorax, emphysema, respiratory failure)	J80, J81, J82, J83, J84, J85, J86, J90, J91, J92, J93, J94, J95, J96, J97, J98, J99	510, 511, 512, 513, 514, 515, 516, 517, 518, 519	17	No (combined/ grouped due to low prevalence, resulting group too general/ unspecific)	
					**Rhinitis**	Vasomotor and allergic rhinitis	J30	477	53	Yes	
**Digestive Conditions**	Conditions of the digestive system	K00-K93	520-579	Yes	**AnusRectum Conditions**	Conditions of anus/rectum (hemorrhage, hemorrhoids, fissure, fistula, abscess, prolapse)	K60, K61, K62, K64	565, 566, 569.0, 569.1,	8	Yes	

								569.2, 569.3, 569.4			
					**Appendicitis**	Appendicitis	K35, K36, K37	540, 541, 542, 543	7	Yes	
					BiliarySystem Conditions	Conditions of liver (e.g., hepatitis), gallbladder, biliary tract, pancreas	K70, K71, K72, K73, K74, K75, K76, K77, K83	570, 571, 572, 573, 574, 575, 576, 577	<5	No (low prevalence)	
					**CeliacDisease**	Celiac disease and intestinal malabsorption	K90	579	16	Yes	
					**Constipation**	Constipation	K59.0	564.0	87	Yes	
					**Dentofacial Anomalies**	Dentofacial anomalies (e.g., teeth [position], caries, embedded/impacted teeth, jaws)	K01, K02, K03, K07, K10	520, 521, 522, 523, 524, 525, 526	8	Yes	
					**Diarrhea**	Functional diarrhea	K59.1	564.5	8	Yes	
					**Dyspepsia**	Functional dyspepsia	K30	536.8	6	Yes	
					**Gastrointestinal Bleeding**	Hematemesis, gastrointestinal hemorrhage, melena	K92.0, K92.1, K92.2	578	6	Yes	
					**GERD**	Gastro-esophageal reflux disorder	K21	530.1, 530.8	32	Yes	
					**Hernia**	Hernia (e.g., inguinal, umbilical, diaphragmatic)	K40, K41, K42, K43, K44, K45, K46	550, 551, 552, 553	40	Yes	
					**IBS**	Irritable bowel syndrome and other/ unspecified functional intestinal conditions	K58, K59.8, K59.9	564.1, 564.8, 564.9	6	Yes	
					**Ileus**	Ileus (paralytic)	K56	560.1	6	Yes	
					**Noninfective IBD**	Crohn's disease and ulcerative colitis (Inflammatory Bowel Disease/IBD)	K50, K51	555, 556	33	Yes	Related conditions, combined due to low prevalence
						Noninfective (allergic/dietetic/food hypersensitivity) gastroenteritis/colitis excl. Crohn's disease or ulcerative colitis	K52.2, K52.8, K52.9	558.3			
					**OtherUpper Gastrointest**	Conditions of esophagus, stomach, and duodenum (inflammation, ulcer, perforation, fistula, dyskinesia; excl. GERD)	K20, K22, K26, K29, K31	530, 531, 532, 533, 534, 535, 537, 538, excl. 530.1/530.8	19	Yes	
					**Stomatitis**	Stomatitis and oral mucositis, oral mucosa lesions (incl. biting), oral cyst, disorders of tongue and salivary gland	K09, K11, K12, K13.1, K13.7, K14	527, 528, 529	16	Yes	
Dermatologic Conditions	Conditions of the skin and subcutaneous tissue	See condition-level for specification	See condition-level for specification	No (only selected conditions examined)	**Bullous Dermatitis**	Bullous disorders incl. pemphigus/ pemphigoid	L10, L11, L12, L13, L14	694	7	Yes	
					**OtherDermatitis Eczema**	Atopic dermatitis (incl. diaper/napkin dermatitis)	L20	691	68	Yes	Related conditions, combined due to low prevalence
						Seborrheic dermatitis	L21	690.1			

						Other/unspecified dermatitis/eczema (incl. infective dermatitis, dermatitis due to in-/ external substances, [allergic] contact dermatitis, diaper/napkin dermatitis)	L22, L23, L24, L25, L26, L27, L30	681.0, 692, 693			
						Lichen and Pruritis	L28, L29	697, 698			
					**Psoriasis**	Psoriasis and other papulosquamous disorders	L40, L41, L42, L43, L44, L45	696	7	Yes	
					**Urticaria**	Urticaria	L50	708	27	Yes	
Musculo-skeletal Conditions	Conditions of the musculo-skeletal system and connective tissue	See condition-level for specification	See condition-level for specification	No (only selected conditions examined)	JuvenileArthritis	Juvenile arthritis	M08	714.3	<5	No (low prevalence)	
Genito-urinary Conditions	Conditions of the genitourinary system	See condition-level for specification	See condition-level for specification	No (only selected conditions examined)	Menstruation Problems	Impaired menstruation (irregularities, pain)	N91, N92, N93, N94	625, 626	5	No (female only)	
					Nephrotic Syndrome	Nephrotic syndrome	N04	581	<5	No (low prevalence)	
**Perinatal Conditions**	Certain conditions originating in the perinatal period	P00-P96	760-779	Yes	**FetalGrowth Retardation**	Conditions related to gestation length, fetal growth/malnutrition	P05, P07	764, 765	177	Yes	
					**Maternal Complications**	Maternal complications (during pregnancy, labor, delivery)	P00, P01, P02, P03, P04	760, 761, 762, 763	48	Yes	
					**Neonatal Jaundice**	Neonatal jaundice	P58, P59	774	91	Yes	
					**PerinatCNS Conditions**	Perinatal intracranial hemorrhage (nontraumatic) and injury to central nervous system (traumatic)	P10, P11, P52	767.0, 767.4, 767.5, 767.7, 772.1, 772.2	16	Yes	Related conditions, combined due to low prevalence
						Convulsions, disturbances of cerebral status	P90, P91	779.0, 779.1, 779.2			
					**PerinatEndo-crineMetabolic**	Perinatal endocrine and metabolic conditions (e.g., hypoglycemia)	P70, P71, P72, P73, P74	775	41	Yes	
					**PerinatFeeding**	Feeding problems of the newborn	P92	779.3	21	Yes	
					**Perinat Hematologic**	Perinatal hematological/hemolytic conditions (e.g., anemia)	P54, P55, P61	776	22	Yes	
					**Perinat Infections**	Perinatal infections (e.g., sepsis, skin infections)	P35, P36, P37, P38, P39	771	29	Yes	
					**PerinatRespirat Cardiovasc**	Perinatal respiratory and cardiovascular conditions (e.g., birth asphyxia, acute respiratory distress syndrome, pneumothorax, persistent fetal circulation)	P20, P21, P22, P23, P24, P25, P26, P27, P28, P29	768, 769, 770	75	Yes	
**Congenital Conditions**	Congenital malformations, deformations,	Q00-Q99	740-759	Yes	**Chromosomal Abnorm**	Chromosomal abnormalities	Q90, Q91, Q92, Q93, Q94, Q95, Q96, Q97, Q98, Q99	758	10	Yes	

	chromosomal abnormalities				**Circulatory SystemMalform**	Malformations of the circulatory system (e.g., atrial/ventricular septal defect, patent ductus arteriosus)	Q20, Q21, Q22, Q23, Q24, Q25, Q26, Q27, Q28	745, 746, 747	40	Yes	
					**Digestive SystemMalform**	Cleft lip/palate	Q35, Q36, Q37	749	26	Yes	Related conditions, combined due to low prevalence
						Malformations of the digestive system (e.g., ankyloglossia)	Q38, Q39, Q40, Q41, Q42, Q43, Q44, Q45	750, 751			
					**GenitalMalform**	Malformations of genitals	Q50, Q51, Q52, Q53, Q54, Q55, Q56	752	19	Yes	
					**HeadMalform**	Malformations of eye, ear, face, and neck	Q10, Q11, Q12, Q13, Q14, Q15, Q16, Q17, Q18	743, 744	17	Yes	
					**Musculoskeletal Malform**	Musculoskeletal malformations	Q65, Q66, Q67, Q68, Q69, Q70, Q71, Q72, Q73, Q74, Q75, Q76, Q77, Q78, Q79	754, 755, 756	44	Yes	
					**NervousSystem Malform**	Malformations of the nervous system (e.g., hydrocephalus, spina bifida)	Q00, Q01, Q02, Q03, Q04, Q05, Q06, Q07	741, 742	20	Yes	
					Respiratory SystemMalform	Malformations of the respiratory system	Q30, Q31, Q32, Q33, Q34	748	<5	No (low prevalence)	
					**UrinarySystem Malform**	Malformations of the urinary system	Q60, Q61, Q62, Q63, Q64	753	11	Yes	
Symptoms	Symptoms, signs, and abnormal clinical and laboratory findings	See condition-level for specification	See condition-level for specification	No (only selected conditions examined)	**Dysphagia**	Dysphagia	R13	787.2	25	Yes	
					**FebrileSeizures**	Febrile seizures/convulsions	R56.0	780.3	14	Yes	
					**NauseaVomiting**	Nausea and vomiting	R11	787.0, 536.2	45	Yes	
					Physiological Development	Lack of expected normal physiological development (delayed milestones, e.g., late talker, late walker)	R62.0, R62.9	n/a	<5	No (low prevalence)	
Trauma	Injury and other consequences of external causes	See condition-level for specification	See condition-level for specification	No (only selected conditions examined)	**Intracranial Injury**	Intracranial injury (e.g., concussion)	S06	850, 851, 852, 853, 854	41	Yes	

	**Conditions grouped across ICD-chapters**						**Comments**
	**Short label**	**Description**	**ICD-10-codes**	**ICD-9-codes**	n_{ARFID}	**Included as outcome?**	
Multiple ICD-chapters	**Allergic Conditions**	Allergic rhinitis, asthma, allergic gastroenteritis, allergic contact dermatitis, urticaria	J30, J45, J46, K52.2, L23, L50	477, 493, 558.3, 692.9, 708	170	Yes	Food allergies were not included (specific ICD-codes could not be ordered from

							Swedish Twin Registry)
	**Autoimmune Conditions**	Autoimmune thyroiditis, diabetes mellitus type I, Crohn's disease, ulcerous colitis, bullous skin conditions (incl. pemphigus/pemphigoid), atopic dermatitis, psoriasis, juvenile arthritis	E06.3, E10, K50, K51, L10, L11, L12, L13, L14, L20, L40, L41, L42, L43, L44, L45, M08	245.2, 250.0, 555, 556, 691, 694, 696, 3	58	Yes	
	**PANS/PANDAS**	Pediatric acute-onset neuropsychiatric syndrome (PANS) including the PANS subgroup related to streptococcal infections (PANDAS)	B95.0, D89.8, D89.9, G96.8	041.0, 279.8, 279.9, 349.8	<5	No (low prevalence)	ICD-codes most used by clinicians comprise single codes from ICD-chapters B (infectious diseases), D (diseases of blood and blood-forming organs involving immune mechanism), and G (nervous system diseases)

Analyzed/included conditions (ICD-chapters [1 digit], individual and grouped conditions within ICD-chapters [3-4 digits], grouped conditions across ICD-chapters [allergic and autoimmune conditions]) are highlighted in **bold**. Short labels of conditions within/across ICD-chapters are sorted in alphabetic order. Some conditions were combined/grouped for context or due to low prevalence (n<5, privacy reasons) of individual conditions (see comments column). *Abbreviations: ADHD, attention deficit hyperactivity disorder; ARFID, avoidant restrictive food intake disorder; CNS, central nervous system; GERD, gastro-esophageal reflux disorder; IBD, inflammatory bowel disease; IBS, irritable bowel syndrome; ICD, International Classification of Diseases.*

Table S3. Prevalence of mental and somatic conditions in ARFID and controls overall, per sex, and per age group

**Condition** n (%)	**A. Overall**		**B. Per sex**				**C. Per age group**					
	**ARFID**	**Controls**	**ARFID**		**Controls**		**ARFID**			**Controls**		
			**Female**	**Male**	**Female**	**Male**	**<6 years**	**6 to <12 years**	**12 to <18 years**	**<6 years**	**6 to <12 years**	**12 to <18 years**
ChapterE:EndocrineConditions	109 (17.69%)	2,119 (7.02%)	52 (21.58%)	57 (15.20%)	1,039 (6.96%)	1,080 (7.08%)	56 (9.09%)	40 (7.14%)	13 (3.32%)	853 (2.83%)	746 (2.54%)	520 (2.28%)
E:ShortStature	28 (4.55%)	423 (1.40%)	18 (7.47%)	10 (2.67%)	183 (1.23%)	240 (1.57%)	n/a	n/a	n/a	n/a	n/a	n/a
E:VolumeDepletion	24 (3.90%)	397 (1.32%)	8 (3.32%)	16 (4.27%)	188 (1.26%)	209 (1.37%)	n/a	n/a	n/a	n/a	n/a	n/a
E:Obesity	7 (1.14%)	351 (1.16%)	n/a	n/a	n/a	n/a	n/a	n/a	n/a	n/a	n/a	n/a
E:PubertyOnset	11 (1.79%)	224 (0.74%)	6 (2.49%)	5 (1.33%)	111 (0.74%)	113 (0.74%)	n/a	n/a	n/a	n/a	n/a	n/a
E:LactoseIntolerance	11 (1.79%)	214 (0.71%)	n/a	n/a	n/a	n/a	n/a	n/a	n/a	n/a	n/a	n/a
E:DiabetesMellitus	5 (0.81%)	190 (0.63%)	n/a	n/a	n/a	n/a	n/a	n/a	n/a	n/a	n/a	n/a
E:ThyroidConditions	7 (1.14%)	161 (0.53%)	n/a	n/a	n/a	n/a	n/a	n/a	n/a	n/a	n/a	n/a
E:PituitaryConditions	9 (1.46%)	76 (0.25%)	n/a	n/a	n/a	n/a	n/a	n/a	n/a	n/a	n/a	n/a
ChapterF:MentalConditions	221 (35.88%)	3,396 (11.25%)	73 (30.29%)	148 (39.47%)	1,422 (9.53%)	1,974 (12.94%)	65 (10.55%)	117 (21.23%)	39 (11.78%)	588 (1.95%)	1,395 (4.71%)	1,413 (6.27%)
F:ADHD	108 (17.53%)	1,168 (3.87%)	33 (13.69%)	75 (20.00%)	356 (2.39%)	812 (5.32%)	9 (1.46%)	70 (11.53%)	29 (7.14%)	39 (0.13%)	556 (1.84%)	573 (2.43%)
F:EnuresisEncopresis	17 (2.76%)	666 (2.21%)	n/a	n/a	n/a	n/a	n/a	n/a	n/a	n/a	n/a	n/a
F:OtherAnxietyConditions	20 (3.25%)	469 (1.55%)	7 (2.90%)	13 (3.47%)	315 (2.11%)	154 (1.01%)	n/a	n/a	n/a	n/a	n/a	n/a
F:Depression	16 (2.60%)	455 (1.51%)	n/a	n/a	n/a	n/a	n/a	n/a	n/a	n/a	n/a	n/a
F:Autism	85 (13.80%)	448 (1.48%)	25 (10.37%)	60 (16.00%)	136 (0.91%)	312 (2.04%)	22 (3.57%)	48 (8.08%)	15 (3.65%)	60 (0.20%)	180 (0.60%)	208 (0.87%)
F:SpeechDevelopment	25 (4.06%)	342 (1.13%)	7 (2.90%)	18 (4.80%)	118 (0.79%)	224 (1.47%)	n/a	n/a	n/a	n/a	n/a	n/a
F:IntellectualDisability	56 (9.09%)	282 (0.93%)	16 (6.64%)	40 (10.67%)	94 (0.63%)	188 (1.23%)	22 (3.57%)	25 (4.21%)	9 (2.12%)	63 (0.21%)	129 (0.43%)	90 (0.38%)
F:OtherBehaviorEmotion	14 (2.27%)	206 (0.68%)	n/a	n/a	n/a	n/a	n/a	n/a	n/a	n/a	n/a	n/a
F:ScholastDevelopment	13 (2.11%)	178 (0.59%)	n/a	n/a	n/a	n/a	n/a	n/a	n/a	n/a	n/a	n/a
F:ObsessCompulsDisorder	5 (0.81%)	149 (0.49%)	n/a	n/a	n/a	n/a	n/a	n/a	n/a	n/a	n/a	n/a
F:TicDisorders	14 (2.27%)	132 (0.44%)	n/a	n/a	n/a	n/a	n/a	n/a	n/a	n/a	n/a	n/a
F:ConductDisorder	9 (1.46%)	124 (0.41%)	n/a	n/a	n/a	n/a	n/a	n/a	n/a	n/a	n/a	n/a
F:MotorDevelopment	18 (2.92%)	110 (0.36%)	9 (3.73%)	9 (2.40%)	38 (0.25%)	72 (0.47%)	n/a	n/a	n/a	n/a	n/a	n/a
F:Phobias	5 (0.81%)	81 (0.27%)	n/a	n/a	n/a	n/a	n/a	n/a	n/a	n/a	n/a	n/a
F:OtherDevelopment	15 (2.44%)	57 (0.19%)	5 (2.07%)	10 (2.67%)	25 (0.17%)	32 (0.21%)	n/a	n/a	n/a	n/a	n/a	n/a
ChapterI:CirculatoryConditions	25 (4.06%)	639 (2.12%)	6 (2.49%)	19 (5.07%)	270 (1.81%)	369 (2.42%)	9 (1.46%)	11 (1.81%)	5 (1.12%)	185 (0.61%)	213 (0.71%)	241 (1.01%)
I:VascVenousLymphatic	7 (1.14%)	260 (0.86%)	n/a	n/a	n/a	n/a	n/a	n/a	n/a	n/a	n/a	n/a
I:HeartPulmonary	12 (1.95%)	205 (0.68%)	n/a	n/a	n/a	n/a	n/a	n/a	n/a	n/a	n/a	n/a
I:VascArterial	6 (0.97%)	148 (0.49%)	n/a	n/a	n/a	n/a	n/a	n/a	n/a	n/a	n/a	n/a
ChapterJ:RespiratoryConditions	327 (53.08%)	11,987 (39.72%)	128 (53.11%)	199 (53.07%)	5,435 (36.42%)	6,552 (42.94%)	259 (42.05%)	48 (13.45%)	20 (8.26%)	8,756 (29.01%)	2,149 (10.03%)	1,082 (6.72%)
J:AcuteUpperRespiratory	187 (30.36%)	6,139 (20.34%)	72 (29.88%)	115 (30.67%)	2,796 (18.74%)	3,343 (21.91%)	152 (24.68%)	25 (5.39%)	10 (2.89%)	5,002 (16.57%)	761 (3.02%)	376 (1.87%)
J:Asthma	128 (20.78%)	3,879 (12.85%)	41 (17.01%)	87 (23.20%)	1,545 (10.35%)	2,334 (15.30%)	89 (14.45%)	31 (5.88%)	8 (2.12%)	2,548 (8.44%)	934 (3.38%)	397 (1.85%)
J:ChronicUpperRespiratory	94 (15.26%)	3,570 (11.83%)	44 (18.26%)	50 (13.33%)	1,599 (10.72%)	1,971 (12.92%)	58 (9.42%)	27 (4.84%)	9 (2.24%)	1,903 (6.31%)	1,123 (3.97%)	544 (2.50%)
J:AcuteLowerRespiratory	112 (18.18%)	3,294 (10.91%)	39 (16.18%)	73 (19.47%)	1,514 (10.15%)	1,780 (11.67%)	n/a	n/a	n/a	n/a	n/a	n/a
J:Rhinitis	53 (8.60%)	1,709 (5.66%)	21 (8.71%)	32 (8.53%)	659 (4.42%)	1,050 (6.88%)	8 (1.30%)	30 (4.93%)	15 (3.46%)	273 (0.90%)	901 (3.01%)	535 (2.32%)

**Condition** n (%)	**A. Overall**		**B. Per sex**				**C. Per age group**					
	**ARFID**	**Controls**	**ARFID**		**Controls**		**ARFID**			**Controls**		
			**Female**	**Male**	**Female**	**Male**	**<6 years**	**6 to <12 years**	**12 to <18 years**	**<6 years**	**6 to <12 years**	**12 to <18 years**
J:ChronicLowerRespiratory	8 (1.30%)	73 (0.24%)	n/a	n/a	n/a	n/a	n/a	n/a	n/a	n/a	n/a	n/a
ChapterK:DigestiveConditions	210 (34.09%)	5,462 (18.10%)	80 (33.20%)	130 (34.67%)	2,430 (16.28%)	3,032 (19.87%)	123 (19.97%)	62 (12.58%)	25 (7.58%)	2,992 (9.91%)	1,499 (5.51%)	971 (4.69%)
K:Constipation	87 (14.12%)	1,893 (6.27%)	37 (15.35%)	50 (13.33%)	945 (6.33%)	948 (6.21%)	40 (6.49%)	37 (6.42%)	10 (2.42%)	944 (3.13%)	722 (2.47%)	227 (0.99%)
K:Hernia	40 (6.49%)	1,260 (4.18%)	6 (2.49%)	34 (9.07%)	258 (1.73%)	1,002 (6.57%)	n/a	n/a	n/a	n/a	n/a	n/a
K:NoninfectiveIBD	33 (5.36%)	613 (2.03%)	13 (5.39%)	20 (5.33%)	275 (1.84%)	338 (2.22%)	18 (2.92%)	10 (1.67%)	5 (1.13%)	394 (1.31%)	125 (0.42%)	94 (0.40%)
K:Appendicitis	7 (1.14%)	517 (1.71%)	n/a	n/a	n/a	n/a	n/a	n/a	n/a	n/a	n/a	n/a
K:CeliacDisease	16 (2.60%)	393 (1.30%)	7 (2.90%)	9 (2.40%)	255 (1.71%)	138 (0.90%)	n/a	n/a	n/a	n/a	n/a	n/a
K:Stomatitis	16 (2.60%)	261 (0.86%)	5 (2.07%)	11 (2.93%)	132 (0.88%)	129 (0.85%)	n/a	n/a	n/a	n/a	n/a	n/a
K:DentofacialAnomalies	8 (1.30%)	247 (0.82%)	n/a	n/a	n/a	n/a	n/a	n/a	n/a	n/a	n/a	n/a
K:GERD	32 (5.19%)	223 (0.74%)	13 (5.39%)	19 (5.07%)	108 (0.72%)	115 (0.75%)	n/a	n/a	n/a	n/a	n/a	n/a
K:OtherUpperGastrointest	19 (3.08%)	206 (0.68%)	8 (3.32%)	11 (2.93%)	118 (0.79%)	88 (0.58%)	n/a	n/a	n/a	n/a	n/a	n/a
K:AnusRectumConditions	8 (1.30%)	191 (0.63%)	n/a	n/a	n/a	n/a	n/a	n/a	n/a	n/a	n/a	n/a
K:Diarrhea	8 (1.30%)	159 (0.53%)	n/a	n/a	n/a	n/a	n/a	n/a	n/a	n/a	n/a	n/a
K:IBS	6 (0.97%)	149 (0.49%)	n/a	n/a	n/a	n/a	n/a	n/a	n/a	n/a	n/a	n/a
K:GastrointestinalBleeding	6 (0.97%)	96 (0.32%)	n/a	n/a	n/a	n/a	n/a	n/a	n/a	n/a	n/a	n/a
K:Ileus	6 (0.97%)	66 (0.22%)	n/a	n/a	n/a	n/a	n/a	n/a	n/a	n/a	n/a	n/a
K:Dyspepsia	6 (0.97%)	58 (0.19%)	n/a	n/a	n/a	n/a	n/a	n/a	n/a	n/a	n/a	n/a
ChapterP:PerinatalConditions	212 (34.42%)	8,538 (28.29%)	78 (32.37%)	134 (35.73%)	4,094 (27.44%)	4,444 (29.13%)	n/a	n/a	n/a	n/a	n/a	n/a
P:FetalGrowthRetardation	177 (28.73%)	7,111 (23.56%)	69 (28.63%)	108 (28.80%)	3,486 (23.36%)	3,625 (23.76%)	n/a	n/a	n/a	n/a	n/a	n/a
P:NeonatalJaundice	91 (14.77%)	3,322 (11.01%)	35 (14.52%)	56 (14.93%)	1,565 (10.49%)	1,757 (11.52%)	n/a	n/a	n/a	n/a	n/a	n/a
P:PerinatRespiratCardiovasc	75 (12.18%)	2,787 (9.23%)	26 (10.79%)	49 (13.07%)	1,216 (8.15%)	1,571 (10.30%)	n/a	n/a	n/a	n/a	n/a	n/a
P:MaternalComplications	48 (7.79%)	2,225 (7.37%)	17 (7.05%)	31 (8.27%)	1,104 (7.40%)	1,121 (7.35%)	n/a	n/a	n/a	n/a	n/a	n/a
P:PerinatEndocrineMetabolic	41 (6.66%)	1,485 (4.92%)	18 (7.47%)	23 (6.13%)	657 (4.40%)	828 (5.43%)	n/a	n/a	n/a	n/a	n/a	n/a
P:PerinatInfections	29 (4.71%)	776 (2.57%)	11 (4.56%)	18 (4.80%)	371 (2.49%)	405 (2.65%)	n/a	n/a	n/a	n/a	n/a	n/a
P:PerinatHematologic	22 (3.57%)	769 (2.55%)	7 (2.90%)	15 (4.00%)	370 (2.48%)	399 (2.62%)	n/a	n/a	n/a	n/a	n/a	n/a
P:PerinatFeeding	21 (3.41%)	483 (1.60%)	8 (3.32%)	13 (3.47%)	257 (1.72%)	226 (1.48%)	n/a	n/a	n/a	n/a	n/a	n/a
P:PerinatCNSConditions	16 (2.60%)	188 (0.62%)	6 (2.49%)	10 (2.67%)	80 (0.54%)	108 (0.71%)	n/a	n/a	n/a	n/a	n/a	n/a
ChapterQ:CongenitalConditions	138 (22.40%)	3,608 (11.96%)	57 (23.65%)	81 (21.60%)	1,470 (9.85%)	2,138 (14.01%)	108 (17.53%)	17 (3.35%)	13 (3.42%)	2,614 (8.66%)	629 (2.28%)	365 (1.68%)
Q:MusculoskeletalMalform	44 (7.14%)	1,038 (3.44%)	16 (6.64%)	28 (7.47%)	484 (3.24%)	554 (3.63%)	25 (4.06%)	13 (2.20%)	6 (1.37%)	659 (2.18%)	197 (0.67%)	182 (0.78%)
Q:CirculatorySystemMalform	40 (6.49%)	843 (2.79%)	21 (8.71%)	19 (5.07%)	451 (3.02%)	392 (2.57%)	n/a	n/a	n/a	n/a	n/a	n/a
Q:GenitalMalform	19 (3.08%)	607 (2.01%)	n/a	n/a	n/a	n/a	n/a	n/a	n/a	n/a	n/a	n/a
Q:DigestiveSystemMalform	26 (4.22%)	491 (1.63%)	11 (4.56%)	15 (4.00%)	158 (1.06%)	333 (2.18%)	n/a	n/a	n/a	n/a	n/a	n/a
Q:HeadMalform	17 (2.76%)	409 (1.36%)	7 (2.90%)	10 (2.67%)	183 (1.23%)	226 (1.48%)	n/a	n/a	n/a	n/a	n/a	n/a
Q:UrinarySystemMalform	11 (1.79%)	184 (0.61%)	n/a	n/a	n/a	n/a	n/a	n/a	n/a	n/a	n/a	n/a
Q:NervousSystemMalform	20 (3.25%)	86 (0.28%)	9 (3.73%)	11 (2.93%)	46 (0.31%)	40 (0.26%)	n/a	n/a	n/a	n/a	n/a	n/a
Q:ChromosomalAbnorm	10 (1.62%)	52 (0.17%)	5 (2.07%)	5 (1.33%)	23 (0.15%)	29 (0.19%)	n/a	n/a	n/a	n/a	n/a	n/a

**Condition** n (%)	**A. Overall**		**B. Per sex**				**C. Per age group**					
	**ARFID**	**Controls**	**ARFID**		**Controls**		**ARFID**			**Controls**		
			**Female**	**Male**	**Female**	**Male**	**<6 years**	**6 to <12 years**	**12 to <18 years**	**<6 years**	**6 to <12 years**	**12 to <18 years**
FurtherConditions												
L:OtherDermatitisEczema	68 (11.04%)	2,284 (7.57%)	28 (11.62%)	40 (10.67%)	1,142 (7.65%)	1,142 (7.49%)	41 (6.66%)	12 (2.09%)	15 (3.53%)	1,202 (3.98%)	725 (2.50%)	357 (1.58%)
S:IntracranialInjury	41 (6.66%)	1,761 (5.84%)	12 (4.98%)	29 (7.73%)	797 (5.34%)	964 (6.32%)	21 (3.41%)	14 (2.35%)	6 (1.38%)	689 (2.28%)	617 (2.09%)	455 (1.98%)
L:Urticaria	27 (4.38%)	900 (2.98%)	8 (3.32%)	19 (5.07%)	421 (2.82%)	479 (3.14%)	n/a	n/a	n/a	n/a	n/a	n/a
R:NauseaVomiting	45 (7.31%)	649 (2.15%)	18 (7.47%)	27 (7.20%)	320 (2.14%)	329 (2.16%)	n/a	n/a	n/a	n/a	n/a	n/a
R:FebrileSeizures	14 (2.27%)	587 (1.95%)	5 (2.07%)	9 (2.40%)	263 (1.76%)	324 (2.12%)	n/a	n/a	n/a	n/a	n/a	n/a
G:Epilepsy	36 (5.84%)	318 (1.05%)	8 (3.32%)	28 (7.47%)	150 (1.01%)	168 (1.10%)	n/a	n/a	n/a	n/a	n/a	n/a
G:CerebralPalsy	25 (4.06%)	202 (0.67%)	7 (2.90%)	18 (4.80%)	94 (0.63%)	108 (0.71%)	n/a	n/a	n/a	n/a	n/a	n/a
L:Psoriasis	7 (1.14%)	186 (0.62%)	n/a	n/a	n/a	n/a	n/a	n/a	n/a	n/a	n/a	n/a
L:BullousDermatitis	7 (1.14%)	170 (0.56%)	n/a	n/a	n/a	n/a	n/a	n/a	n/a	n/a	n/a	n/a
R:Dysphagia	25 (4.06%)	120 (0.40%)	6 (2.49%)	19 (5.07%)	63 (0.42%)	57 (0.37%)	12 (1.95%)	7 (1.16%)	6 (1.33%)	47 (0.16%)	48 (0.16%)	25 (0.10%)
EntireChapterC:Cancer	13 (2.11%)	79 (0.26%)	5 (2.07%)	8 (2.13%)	29 (0.19%)	50 (0.33%)	n/a	n/a	n/a	n/a	n/a	n/a
GroupedConditions												
AllergicConditions	170 (27.60%)	5,366 (17.78%)	59 (24.48%)	111 (29.60%)	2,244 (15.04%)	3,122 (20.46%)	105 (17.05%)	45 (8.81%)	20 (5.59%)	3,110 (10.31%)	1,513 (5.59%)	743 (3.60%)
AutoimmuneConditions	58 (9.42%)	2,218 (7.35%)	28 (11.62%)	30 (8.00%)	1,087 (7.28%)	1,131 (7.41%)	34 (5.52%)	12 (2.06%)	12 (2.81%)	1,133 (3.75%)	689 (2.37%)	396 (1.75%)

Absolute number (n) and percentage of individuals with ARFID and controls are stated for each condition overall (A.), per sex (B.), and per age group (C.). Conditions are sorted in descending order of overall prevalence (A.) in controls within ICD-chapters, the group of further conditions, and grouped allergic and autoimmune conditions (highlighted with blue background). Conditions with n<5 individuals in ARFID or controls are not reported for privacy reasons (n/a). *Abbreviations: ADHD, attention deficit hyperactivity disorder; ARFID, avoidant restrictive food intake disorder; CNS, central nervous system; GERD, gastro-esophageal reflux disorder; IBD, inflammatory bowel disease; IBS, irritable bowel syndrome.*

Table S4. Base Cox regression model estimates in ARFID vs. controls

Condition	HR	95%CI LL	95%CI UL	Robust SE	z statistic	p-value	Significant (*) after FDR/BH
ChapterE:EndocrineConditions	2.73	2.21	3.36	0.11	9.45	<0.0001	*
E:PituitaryConditions	5.56	2.74	11.30	0.36	4.75	<0.0001	*
E:ShortStature	3.29	2.16	5.03	0.22	5.52	<0.0001	*
E:VolumeDepletion	2.78	1.81	4.28	0.22	4.66	<0.0001	*
E:PubertyOnset	2.65	1.38	5.09	0.33	2.92	0.003	*
E:LactoseIntolerance	2.52	1.30	4.89	0.34	2.74	0.006	*
E:ThyroidConditions	2.43	1.14	5.21	0.39	2.29	0.022	*
E:DiabetesMellitus	1.33	0.54	3.25	0.46	0.62	0.532	
E:Obesity	0.98	0.47	2.05	0.38	-0.06	0.955	
ChapterF:MentalConditions	3.90	3.36	4.52	0.08	17.95	<0.0001	*
F:OtherDevelopment	12.49	7.03	22.21	0.29	8.60	<0.0001	*
F:IntellectualDisability	10.27	7.59	13.90	0.15	15.09	<0.0001	*
F:Autism	9.68	7.52	12.47	0.13	17.60	<0.0001	*
F:MotorDevelopment	7.41	4.33	12.68	0.27	7.30	<0.0001	*
F:ADHD	4.86	3.93	6.00	0.11	14.69	<0.0001	*
F:TicDisorders	4.80	2.64	8.71	0.30	5.15	<0.0001	*
F:ScholastDevelopment	3.58	2.04	6.30	0.29	4.43	<0.0001	*
F:ConductDisorder	3.58	1.84	6.96	0.34	3.76	0.0002	*
F:Phobias	3.57	1.46	8.74	0.46	2.78	0.005	*
F:OtherBehaviorEmotion	3.42	2.00	5.85	0.27	4.49	<0.0001	*
F:SpeechDevelopment	3.32	2.18	5.05	0.21	5.60	<0.0001	*
F:OtherAnxietyConditions	2.44	1.56	3.81	0.23	3.91	<0.0001	*
F:Depression	2.13	1.28	3.52	0.26	2.93	0.003	*
F:ObsessCompulsDisorder	1.80	0.73	4.40	0.46	1.28	0.200	
F:EnuresisEncopresis	1.11	0.69	1.79	0.24	0.42	0.676	
ChapterI:CirculatoryConditions	1.96	1.29	2.97	0.21	3.17	0.002	*
I:HeartPulmonary	3.02	1.61	5.64	0.32	3.46	0.0005	*
I:VascArterial	2.02	0.89	4.60	0.42	1.68	0.094	
I:VascVenousLymphatic	1.29	0.61	2.73	0.38	0.66	0.512	
ChapterJ:RespiratoryConditions	1.42	1.26	1.60	0.06	5.72	<0.0001	*
J:ChronicLowerRespiratory	4.87	2.35	10.10	0.37	4.25	<0.0001	*
J:AcuteLowerRespiratory	1.59	1.30	1.94	0.10	4.57	<0.0001	*
J:Asthma	1.57	1.30	1.88	0.09	4.74	<0.0001	*
J:Rhinitis	1.52	1.14	2.01	0.14	2.89	0.004	*
J:AcuteUpperRespiratory	1.44	1.24	1.68	0.08	4.68	<0.0001	*
J:ChronicUpperRespiratory	1.30	1.05	1.60	0.11	2.37	0.018	*
ChapterK:DigestiveConditions	2.03	1.76	2.35	0.07	9.58	<0.0001	*
K:GERD	6.74	4.60	9.87	0.19	9.79	<0.0001	*
K:Dyspepsia	5.67	2.44	13.16	0.43	4.04	<0.0001	*
K:OtherUpperGastrointest	4.97	3.10	7.99	0.24	6.64	<0.0001	*
K:Ileus	4.04	1.75	9.35	0.43	3.26	0.001	*
K:Stomatitis	3.02	1.78	5.13	0.27	4.09	<0.0001	*
K:GastrointestinalBleeding	2.97	1.31	6.76	0.42	2.60	0.009	*
K:NoninfectiveIBD	2.58	1.76	3.76	0.19	4.90	<0.0001	*
K:Diarrhea	2.34	1.14	4.79	0.37	2.32	0.020	*
K:Constipation	2.21	1.77	2.77	0.11	6.91	<0.0001	*
K:CeliacDisease	2.11	1.28	3.47	0.26	2.92	0.004	*
K:AnusRectumConditions	2.04	1.00	4.15	0.36	1.97	0.049	
K:IBS	2.00	0.89	4.49	0.41	1.68	0.094	
K:DentofacialAnomalies	1.81	0.89	3.67	0.36	1.64	0.100	
K:Hernia	1.41	1.01	1.96	0.17	2.03	0.042	

Condition	HR	95%CI LL	95%CI UL	Robust SE	z statistic	p-value	Significant (*) after FDR/BH
K:Appendicitis	0.70	0.33	1.48	0.38	-0.93	0.353	
ChapterP:PerinatalConditions	1.23	1.07	1.42	0.07	2.87	0.004	*
P:PerinatCNSConditions	3.99	2.37	6.71	0.26	5.23	<0.0001	*
P:PerinatFeeding	2.04	1.29	3.23	0.23	3.07	0.002	*
P:PerinatInfections	1.80	1.22	2.67	0.20	2.96	0.003	*
P:NeonatalJaundice	1.35	1.09	1.67	0.11	2.76	0.006	*
P:PerinatEndocrineMetabolic	1.33	0.98	1.81	0.16	1.81	0.071	
P:PerinatHematologic	1.33	0.84	2.10	0.23	1.22	0.221	
P:PerinatRespiratCardiovasc	1.30	1.02	1.65	0.12	2.12	0.034	*
P:FetalGrowthRetardation	1.24	1.06	1.45	0.08	2.64	0.008	*
P:MaternalComplications	1.13	0.85	1.50	0.15	0.81	0.417	
ChapterQ:CongenitalConditions	1.89	1.58	2.26	0.09	7.00	<0.0001	*
Q:NervousSystemMalform	11.50	7.06	18.75	0.25	9.80	<0.0001	*
Q:ChromosomalAbnorm	9.21	4.68	18.13	0.35	6.42	<0.0001	*
Q:UrinarySystemMalform	2.68	1.39	5.18	0.34	2.93	0.003	*
Q:DigestiveSystemMalform	2.40	1.62	3.57	0.20	4.34	<0.0001	*
Q:CirculatorySystemMalform	2.35	1.70	3.26	0.17	5.13	<0.0001	*
Q:HeadMalform	2.02	1.24	3.29	0.25	2.84	0.005	*
Q:MusculoskeletalMalform	2.01	1.48	2.73	0.16	4.43	<0.0001	*
Q:GenitalMalform	1.28	0.81	2.03	0.23	1.07	0.286	
FurtherConditions							
R:Dysphagia	10.40	6.79	15.92	0.22	10.77	<0.0001	*
EntireChapterC:Cancer	7.55	4.14	13.77	0.31	6.59	<0.0001	*
G:CerebralPalsy	6.23	4.09	9.47	0.21	8.55	<0.0001	*
G:Epilepsy	5.82	4.12	8.24	0.18	9.96	<0.0001	*
R:NauseaVomiting	3.25	2.40	4.41	0.15	7.62	<0.0001	*
L:BullousDermatitis	2.21	1.04	4.73	0.39	2.05	0.040	
L:Psoriasis	1.95	0.92	4.11	0.38	1.75	0.080	
L:OtherDermatitisEczema	1.46	1.13	1.88	0.13	2.92	0.004	*
L:Urticaria	1.44	0.97	2.13	0.20	1.80	0.072	
S:IntracranialInjury	1.17	0.86	1.60	0.16	0.99	0.323	
R:FebrileSeizures	1.13	0.67	1.92	0.27	0.45	0.651	
GroupedConditions							
AllergicConditions	1.56	1.32	1.83	0.08	5.34	<0.0001	*
AutoimmuneConditions	1.29	0.98	1.70	0.14	1.79	0.073	

Stated are for each condition: hazard ratio in ARFID vs. controls (HR), lower/upper limits of the cluster-robust 95% confidence interval (95% CI LL/UL), cluster-robust standard error (SE), z statistic, raw p-value, and significance according to the false discovery rate-adjusted threshold (FDR/Benjamini-Hochberg procedure [BH]) from the base Cox regression model (predictor: group [ARFID vs. controls]; covariates: sex [female/male], birth year [1992–2008, factorized]; robust sandwich estimates given clustered twin data). Significantly different risks in ARFID vs. controls at α_{FDR}=0.0343 are marked by an asterisk (*). Conditions are sorted in descending order of HRs within ICD-chapters, the group of further conditions, and grouped allergic and autoimmune conditions (highlighted with blue background). *Abbreviations: ADHD, attention deficit hyperactivity disorder; ARFID, avoidant restrictive food intake disorder; CNS, central nervous system; GERD, gastro-esophageal reflux disorder; IBD, inflammatory bowel disease; IBS, irritable bowel syndrome.*

Table S5. Cumulative incidences of mental and somatic conditions before ages 6, 12, and 18 years in ARFID and controls

Condition	Age: <6 years						Age: <12 years						Age: <18 years					
	ARFID			Controls			ARFID			Controls			ARFID			Controls		
	Cum. Inc.	95%CI LL	95%CI UL	Cum. Inc.	95%CI LL	95%CI UL	Cum. Inc.	95%CI LL	95%CI UL	Cum. Inc.	95%CI LL	95%CI UL	Cum. Inc.	95%CI LL	95%CI UL	Cum. Inc.	95%CI LL	95%CI UL
ChapterE:EndocrineConditions	0.0925	0.0679	0.1165	0.0302	0.0258	0.0346	0.1604	0.1282	0.1915	0.0575	0.0514	0.0636	0.1993	0.1616	0.2353	0.0816	0.0736	0.0895
E:PituitaryConditions	0.0049	0.0000	0.0103	0.0005	0.0000	0.0010	0.0121	0.0031	0.0210	0.0023	0.0010	0.0035	0.0183	0.0059	0.0306	0.0023	0.0010	0.0035
E:ShortStature	0.0114	0.0018	0.0208	0.0041	0.0024	0.0057	0.0404	0.0222	0.0581	0.0102	0.0076	0.0129	0.0506	0.0300	0.0708	0.0161	0.0125	0.0197
E:VolumeDepletion	0.0292	0.0151	0.0431	0.0123	0.0095	0.0152	0.0398	0.0234	0.0559	0.0147	0.0115	0.0178	0.0398	0.0234	0.0559	0.0157	0.0123	0.0190
E:PubertyOnset	0.0049	0.0000	0.0119	0.0006	0.0000	0.0013	0.0102	0.0009	0.0194	0.0030	0.0014	0.0045	0.0263	0.0092	0.0431	0.0091	0.0061	0.0120
E:LactoseIntolerance	0.0097	0.0020	0.0175	0.0042	0.0025	0.0059	0.0186	0.0068	0.0303	0.0063	0.0042	0.0083	0.0186	0.0068	0.0303	0.0068	0.0047	0.0090
E:ThyroidConditions	0.0065	0.0001	0.0128	0.0013	0.0004	0.0022	0.0086	0.0010	0.0162	0.0029	0.0015	0.0043	0.0150	0.0030	0.0268	0.0060	0.0036	0.0084
E:DiabetesMellitus	0.0049	0.0000	0.0103	0.0016	0.0006	0.0026	0.0067	0.0001	0.0132	0.0037	0.0021	0.0053	0.0097	0.0009	0.0184	0.0073	0.0046	0.0099
E:Obesity	0.0016	0.0000	0.0048	0.0016	0.0005	0.0027	0.0104	0.0021	0.0187	0.0094	0.0067	0.0120	0.0126	0.0032	0.0220	0.0163	0.0124	0.0201
ChapterF:MentalConditions	0.1071	0.0814	0.1322	0.0235	0.0196	0.0275	0.3081	0.2674	0.3465	0.0854	0.0779	0.0929	0.4159	0.3669	0.4611	0.1549	0.1436	0.1661
F:OtherDevelopment	0.0146	0.0051	0.0240	0.0013	0.0004	0.0022	0.0237	0.0113	0.0359	0.0015	0.0005	0.0024	0.0259	0.0128	0.0388	0.0015	0.0005	0.0024
F:IntellectualDisability	0.0357	0.0203	0.0509	0.0024	0.0012	0.0037	0.0811	0.0576	0.1039	0.0076	0.0053	0.0098	0.1070	0.0770	0.1360	0.0135	0.0101	0.0168
F:Autism	0.0357	0.0197	0.0515	0.0021	0.0010	0.0033	0.1209	0.0912	0.1496	0.0090	0.0065	0.0115	0.1642	0.1281	0.1988	0.0193	0.0151	0.0234
F:MotorDevelopment	0.0162	0.0062	0.0262	0.0028	0.0013	0.0042	0.0285	0.0141	0.0427	0.0047	0.0028	0.0065	0.0309	0.0158	0.0457	0.0049	0.0030	0.0068
F:ADHD	0.0162	0.0062	0.0262	0.0024	0.0010	0.0038	0.1381	0.1078	0.1674	0.0301	0.0254	0.0347	0.2196	0.1780	0.2592	0.0617	0.0540	0.0693
F:TicDisorders	0.0016	0.0000	0.0048	0.0003	0.0000	0.0008	0.0173	0.0057	0.0287	0.0044	0.0026	0.0062	0.0293	0.0127	0.0456	0.0069	0.0044	0.0093
F:ScholastDevelopment	0.0016	0.0000	0.0048	0.0002	0.0000	0.0005	0.0113	0.0023	0.0203	0.0033	0.0018	0.0049	0.0335	0.0146	0.0521	0.0091	0.0062	0.0121
F:ConductDisorder	0.0049	0.0000	0.0103	0.0002	0.0000	0.0005	0.0137	0.0042	0.0231	0.0027	0.0013	0.0041	0.0169	0.0055	0.0281	0.0061	0.0036	0.0085
F:Phobias	0.0016	0.0000	0.0048	0.0003	0.0000	0.0008	0.0076	0.0001	0.0150	0.0007	0.0000	0.0015	0.0114	0.0008	0.0219	0.0030	0.0012	0.0049
F:OtherBehaviorEmotion	0.0049	0.0000	0.0103	0.0011	0.0003	0.0020	0.0188	0.0077	0.0297	0.0041	0.0023	0.0058	0.0290	0.0130	0.0448	0.0099	0.0068	0.0130
F:SpeechDevelopment	0.0227	0.0101	0.0352	0.0073	0.0050	0.0096	0.0350	0.0195	0.0502	0.0116	0.0088	0.0144	0.0450	0.0269	0.0628	0.0148	0.0115	0.0181
F:OtherAnxietyConditions	0.0016	0.0000	0.0048	0.0002	0.0000	0.0005	0.0227	0.0099	0.0353	0.0021	0.0008	0.0033	0.0509	0.0275	0.0736	0.0250	0.0195	0.0306
F:Depression	0.0000	0.0000	0.0000	0.0003	0.0000	0.0008	0.0115	0.0023	0.0206	0.0015	0.0005	0.0026	0.0417	0.0207	0.0623	0.0190	0.0142	0.0238
F:ObsessCompulsDisorder	0.0000	0.0000	0.0000	0.0002	0.0000	0.0005	0.0059	0.0000	0.0126	0.0017	0.0006	0.0028	0.0117	0.0012	0.0221	0.0088	0.0057	0.0119
F:EnuresisEncopresis	0.0032	0.0000	0.0077	0.0055	0.0037	0.0074	0.0265	0.0136	0.0393	0.0291	0.0246	0.0335	0.0294	0.0153	0.0434	0.0298	0.0253	0.0343
ChapterI:CirculatoryConditions	0.0146	0.0051	0.0240	0.0060	0.0041	0.0079	0.0346	0.0195	0.0495	0.0128	0.0099	0.0156	0.0519	0.0293	0.0740	0.0276	0.0226	0.0327
I:HeartPulmonary	0.0081	0.0010	0.0152	0.0028	0.0014	0.0041	0.0117	0.0030	0.0203	0.0045	0.0028	0.0063	0.0294	0.0084	0.0499	0.0086	0.0058	0.0114
I:VascArterial	0.0049	0.0000	0.0104	0.0011	0.0003	0.0020	0.0107	0.0021	0.0193	0.0017	0.0006	0.0027	0.0107	0.0021	0.0193	0.0042	0.0023	0.0062
I:VascVenousLymphatic	0.0032	0.0000	0.0077	0.0021	0.0010	0.0033	0.0102	0.0020	0.0184	0.0055	0.0036	0.0073	0.0135	0.0031	0.0238	0.0121	0.0087	0.0154
ChapterJ:RespiratoryConditions	0.4205	0.3786	0.4595	0.3258	0.3134	0.3380	0.5046	0.4613	0.5445	0.3960	0.3830	0.4087	0.5561	0.5107	0.5973	0.4509	0.4369	0.4645
J:ChronicLowerRespiratory	0.0114	0.0030	0.0197	0.0026	0.0013	0.0039	0.0130	0.0040	0.0219	0.0026	0.0013	0.0039	0.0130	0.0040	0.0219	0.0030	0.0015	0.0045
J:AcuteLowerRespiratory	0.1607	0.1302	0.1902	0.1120	0.1037	0.1203	0.1766	0.1442	0.2077	0.1231	0.1144	0.1316	0.1872	0.1535	0.2197	0.1305	0.1214	0.1395
J:Asthma	0.1461	0.1163	0.1749	0.1050	0.0969	0.1131	0.1996	0.1652	0.2325	0.1379	0.1287	0.1470	0.2240	0.1864	0.2600	0.1613	0.1511	0.1715

Condition	Age: <6 years						Age: <12 years						Age: <18 years					
	ARFID			Controls			ARFID			Controls			ARFID			Controls		
	Cum. Inc.	95%CI LL	95%CI UL	Cum. Inc.	95%CI LL	95%CI UL	Cum. Inc.	95%CI LL	95%CI UL	Cum. Inc.	95%CI LL	95%CI UL	Cum. Inc.	95%CI LL	95%CI UL	Cum. Inc.	95%CI LL	95%CI UL
J:Rhinitis	0.0130	0.0040	0.0219	0.0115	0.0088	0.0143	0.0681	0.0462	0.0894	0.0472	0.0415	0.0528	0.1126	0.0805	0.1437	0.0794	0.0712	0.0875
J:AcuteUpperRespiratory	0.2484	0.2120	0.2831	0.1802	0.1701	0.1902	0.2903	0.2518	0.3269	0.2046	0.1940	0.2151	0.3175	0.2766	0.3560	0.2262	0.2147	0.2375
J:ChronicUpperRespiratory	0.0958	0.0715	0.1194	0.0722	0.0656	0.0789	0.1423	0.1123	0.1712	0.1102	0.1021	0.1182	0.1676	0.1343	0.1997	0.1412	0.1313	0.1510
ChapterK:DigestiveConditions	0.2013	0.1679	0.2334	0.1097	0.1016	0.1178	0.3081	0.2679	0.3462	0.1655	0.1557	0.1752	0.3754	0.3302	0.4175	0.2154	0.2035	0.2272
K:GERD	0.0308	0.0171	0.0444	0.0037	0.0021	0.0053	0.0470	0.0293	0.0645	0.0070	0.0047	0.0092	0.0582	0.0373	0.0787	0.0115	0.0083	0.0147
K:Dyspepsia	0.0016	0.0000	0.0048	0.0000	0.0000	0.0000	0.0098	0.0012	0.0183	0.0005	0.0000	0.0011	0.0124	0.0024	0.0223	0.0031	0.0012	0.0049
K:OtherUpperGastrointest	0.0146	0.0051	0.0240	0.0008	0.0001	0.0015	0.0220	0.0100	0.0338	0.0034	0.0019	0.0049	0.0385	0.0206	0.0560	0.0097	0.0065	0.0129
K:Ileus	0.0049	0.0000	0.0104	0.0024	0.0012	0.0037	0.0086	0.0010	0.0161	0.0024	0.0012	0.0037	0.0119	0.0019	0.0218	0.0028	0.0014	0.0042
K:Stomatitis	0.0162	0.0062	0.0261	0.0045	0.0029	0.0062	0.0243	0.0106	0.0378	0.0072	0.0050	0.0094	0.0295	0.0141	0.0446	0.0094	0.0067	0.0120
K:GastrointestinalBleeding	0.0081	0.0010	0.0152	0.0023	0.0011	0.0035	0.0081	0.0010	0.0152	0.0031	0.0017	0.0045	0.0105	0.0020	0.0189	0.0040	0.0023	0.0058
K:NoninfectiveIBD	0.0292	0.0144	0.0438	0.0156	0.0124	0.0188	0.0471	0.0282	0.0655	0.0195	0.0160	0.0231	0.0607	0.0385	0.0824	0.0253	0.0209	0.0297
K:Diarrhea	0.0097	0.0020	0.0174	0.0041	0.0024	0.0057	0.0115	0.0030	0.0199	0.0055	0.0036	0.0074	0.0137	0.0042	0.0231	0.0077	0.0051	0.0102
K:Constipation	0.0666	0.0457	0.0869	0.0343	0.0296	0.0389	0.1283	0.0995	0.1563	0.0628	0.0565	0.0690	0.1567	0.1232	0.1890	0.0761	0.0688	0.0833
K:CeliacDisease	0.0162	0.0062	0.0261	0.0070	0.0049	0.0091	0.0236	0.0113	0.0358	0.0111	0.0083	0.0138	0.0287	0.0145	0.0426	0.0133	0.0101	0.0166
K:AnusRectumConditions	0.0049	0.0000	0.0104	0.0028	0.0014	0.0041	0.0100	0.0020	0.0179	0.0046	0.0028	0.0063	0.0162	0.0043	0.0279	0.0065	0.0042	0.0087
K:IBS	0.0032	0.0000	0.0077	0.0029	0.0014	0.0044	0.0070	0.0001	0.0139	0.0040	0.0023	0.0057	0.0127	0.0022	0.0232	0.0072	0.0046	0.0098
K:DentofacialAnomalies	0.0016	0.0000	0.0048	0.0006	0.0000	0.0013	0.0054	0.0000	0.0115	0.0029	0.0015	0.0043	0.0197	0.0055	0.0338	0.0133	0.0095	0.0171
K:Hernia	0.0617	0.0420	0.0810	0.0390	0.0340	0.0439	0.0650	0.0443	0.0852	0.0447	0.0394	0.0500	0.0650	0.0443	0.0852	0.0483	0.0426	0.0540
K:Appendicitis	0.0032	0.0000	0.0077	0.0015	0.0005	0.0024	0.0068	0.0001	0.0135	0.0097	0.0071	0.0123	0.0157	0.0035	0.0277	0.0219	0.0173	0.0266
ChapterP:PerinatalConditions	n/a	n/a	n/a	n/a	n/a	n/a	n/a	n/a	n/a	n/a	n/a	n/a	n/a	n/a	n/a	n/a	n/a	n/a
ChapterQ:CongenitalConditions	0.1769	0.1452	0.2075	0.0998	0.0922	0.1074	0.2047	0.1710	0.2371	0.1217	0.1133	0.1300	0.2428	0.2042	0.2795	0.1369	0.1278	0.1460
Q:NervousSystemMalform	0.0325	0.0184	0.0464	0.0024	0.0012	0.0037	0.0325	0.0184	0.0464	0.0028	0.0014	0.0041	0.0325	0.0184	0.0464	0.0030	0.0016	0.0044
Q:ChromosomalAbnorm	0.0114	0.0030	0.0197	0.0013	0.0004	0.0022	0.0130	0.0040	0.0219	0.0013	0.0004	0.0022	0.0207	0.0067	0.0345	0.0013	0.0004	0.0022
Q:UrinarySystemMalform	0.0146	0.0041	0.0250	0.0055	0.0036	0.0074	0.0166	0.0054	0.0277	0.0063	0.0043	0.0084	0.0199	0.0070	0.0327	0.0063	0.0043	0.0084
Q:DigestiveSystemMalform	0.0390	0.0236	0.0541	0.0166	0.0133	0.0198	0.0390	0.0236	0.0541	0.0185	0.0151	0.0219	0.0441	0.0272	0.0608	0.0196	0.0160	0.0231
Q:CirculatorySystemMalform	0.0584	0.0392	0.0773	0.0265	0.0223	0.0306	0.0633	0.0434	0.0828	0.0279	0.0236	0.0322	0.0663	0.0455	0.0866	0.0296	0.0251	0.0340
Q:HeadMalform	0.0195	0.0085	0.0303	0.0076	0.0055	0.0098	0.0264	0.0135	0.0390	0.0125	0.0097	0.0154	0.0289	0.0152	0.0424	0.0136	0.0106	0.0166
Q:MusculoskeletalMalform	0.0422	0.0256	0.0586	0.0260	0.0219	0.0300	0.0637	0.0434	0.0837	0.0327	0.0282	0.0372	0.0805	0.0563	0.1042	0.0419	0.0364	0.0474
Q:GenitalMalform	0.0292	0.0159	0.0424	0.0211	0.0175	0.0247	0.0309	0.0172	0.0444	0.0269	0.0227	0.0310	0.0309	0.0172	0.0444	0.0276	0.0234	0.0318
FurtherConditions																		
R:Dysphagia	0.0195	0.0085	0.0303	0.0011	0.0003	0.0020	0.0320	0.0177	0.0461	0.0038	0.0022	0.0054	0.0485	0.0291	0.0675	0.0050	0.0030	0.0069
EntireChapterC:Cancer	0.0114	0.0030	0.0197	0.0018	0.0007	0.0028	0.0168	0.0064	0.0271	0.0027	0.0014	0.0040	0.0247	0.0110	0.0382	0.0031	0.0016	0.0046
G:CerebralPalsy	0.0406	0.0249	0.0560	0.0044	0.0027	0.0060	0.0406	0.0249	0.0560	0.0052	0.0034	0.0070	0.0406	0.0249	0.0560	0.0054	0.0036	0.0073
G:Epilepsy	0.0373	0.0223	0.0522	0.0044	0.0027	0.0060	0.0535	0.0352	0.0713	0.0098	0.0072	0.0123	0.0640	0.0431	0.0844	0.0127	0.0096	0.0159

Condition	Age: <6 years						Age: <12 years						Age: <18 years					
	ARFID			Controls			ARFID			ARFID			Controls			ARFID		
	Cum. Inc.	95%CI LL	95%CI UL	Cum. Inc.	95%CI LL	95%CI UL	Cum. Inc.	95%CI LL	95%CI UL	Cum. Inc.	95%CI LL	95%CI UL	Cum. Inc.	95%CI LL	95%CI UL	Cum. Inc.	95%CI LL	95%CI UL
R:NauseaVomiting	0.0503	0.0329	0.0675	0.0131	0.0103	0.0160	0.0680	0.0476	0.0880	0.0197	0.0162	0.0233	0.0786	0.0558	0.1008	0.0247	0.0205	0.0290
L:BullousDermatitis	0.0114	0.0030	0.0197	0.0047	0.0029	0.0065	0.0114	0.0030	0.0197	0.0052	0.0033	0.0070	0.0114	0.0030	0.0197	0.0054	0.0035	0.0073
L:Psoriasis	0.0049	0.0000	0.0104	0.0015	0.0005	0.0024	0.0083	0.0010	0.0154	0.0047	0.0029	0.0065	0.0133	0.0033	0.0232	0.0093	0.0064	0.0122
L:OtherDermatitisEczema	0.0666	0.0454	0.0872	0.0464	0.0411	0.0518	0.0879	0.0639	0.1113	0.0759	0.0689	0.0828	0.1268	0.0962	0.1564	0.0964	0.0880	0.1047
L:Urticaria	0.0244	0.0114	0.0372	0.0148	0.0118	0.0178	0.0292	0.0152	0.0431	0.0255	0.0215	0.0295	0.0594	0.0351	0.0830	0.0338	0.0287	0.0389
S:IntracranialInjury	0.0357	0.0210	0.0502	0.0266	0.0226	0.0306	0.0586	0.0395	0.0773	0.0465	0.0411	0.0519	0.0753	0.0521	0.0980	0.0693	0.0619	0.0767
R:FebrileSeizures	0.0195	0.0085	0.0303	0.0185	0.0150	0.0220	0.0213	0.0097	0.0327	0.0187	0.0152	0.0222	0.0243	0.0114	0.0370	0.0193	0.0157	0.0229
GroupedConditions																		
AllergicConditions	0.1721	0.1402	0.2028	0.1252	0.1165	0.1338	0.2496	0.2121	0.2854	0.1775	0.1674	0.1875	0.3071	0.2633	0.3482	0.2217	0.2098	0.2334
AutoimmuneConditions	0.0568	0.0374	0.0758	0.0433	0.0381	0.0485	0.0758	0.0533	0.0977	0.0707	0.0640	0.0774	0.1063	0.0779	0.1338	0.0922	0.0839	0.1004

Cumulative incidence (Cum. Inc., absolute risk) as the probability (proportion) of having any of the analyzed conditions before three consecutive time points (i.e., before 6, 12, and 18 years of age) is stated in ARFID and controls, including lower/upper limits of the cluster-robust 95% confidence interval (95% CI LL/UL). Perinatal conditions were excluded [n/a] given their occurrence during the perinatal period and lack of age-related dynamics). Cumulative incidence was estimated using the Kaplan-Meier method (Cum. Inc. = 1 - Kaplan-Meier survival estimate, accounting for censoring) after creating a modified analysis sample by matching n=10 unexposed individuals to each (n=1) individual twin with ARFID, stratified by sex and birth year. Conditions are sorted in accordance with Table S4, i.e., in descending order of base cox regression model hazard ratios within ICD-chapters, the group of further conditions, and grouped allergic and autoimmune conditions (highlighted with blue background). *Abbreviations: ADHD, attention deficit hyperactivity disorder; ARFID, avoidant restrictive food intake disorder; CNS, central nervous system; GERD, gastro-esophageal reflux disorder; IBD, inflammatory bowel disease; IBS, irritable bowel syndrome.*

Table S6. Number of distinct overall, all mental, and all somatic diagnoses in ARFID vs. controls

Condition	ARFID		Controls		Poisson regression model statistics						
	Mean	SEM	Mean	SEM	IRR	95%CI LL	95%CI UL	Robust SE	z statistic	p-value	Significant (*) after FDR/BH
All diagnoses	6.27	0.28	2.78	0.02	2.18	1.99	2.39	0.05	16.59	<0.0001	*
Mental diagnoses	1.01	0.07	0.22	0.00	4.65	3.99	5.42	0.08	19.73	<0.0001	*
Somatic diagnoses	5.26	0.26	2.56	0.02	1.98	1.79	2.19	0.05	13.55	<0.0001	*

Mean and standard error of the mean (SEM) for number of *all* distinct diagnoses (i.e., unique diagnostic ICD-codes), number of distinct *mental* diagnoses, and number of distinct *somatic* diagnoses per individual twin are given in ARFID and controls. As test statistics, incidence rate ratio in ARFID vs. controls (IRR), lower/upper limits of the cluster-robust 95% confidence interval (95% CI LL/UL), cluster-robust standard error (SE), z statistic, raw p-value, and significance according to false discovery rate-adjusted threshold (FDR/Benjamini-Hochberg procedure [BH]) are stated for all distinct diagnoses, distinct mental diagnoses, and distinct somatic diagnoses. Statistics were obtained with Poisson regression models (predictor: group [ARFID vs. controls]; covariates: sex [female/male], birth year [1992–2008, factorized]; robust sandwich estimates given clustered twin data; offset term to account for follow-up/exposure time). Significantly different incidence rates in ARFID vs. controls at α_{FDR}=0.0343 are marked by an asterisk (*). *Abbreviation: ARFID, avoidant restrictive food intake disorder.*

Table S7. Number of inpatient days due to any, any mental, and any somatic diagnosis in ARFID vs. controls

Condition	ARFID		Controls		Poisson regression model statistics						
	**Mean**	**SEM**	**Mean**	**SEM**	**IRR**	**95%CI LL**	**95%CI UL**	**Robust SE**	**z statistic**	**p-value**	**Significant (*) after FDR/BH**
Any diagnosis	26.61	2.73	9.18	0.13	2.88	2.34	3.55	0.11	9.96	<0.0001	*
Mental diagnosis	1.00	0.58	0.24	0.04	5.50	1.72	17.60	0.59	2.87	0.004	*
Somatic diagnosis	23.03	2.44	8.49	0.12	2.68	2.16	3.32	0.11	8.97	<0.0001	*

Mean and standard error of the mean (SEM) for number of inpatient days per individual twin due to any diagnosis (i.e., unique diagnostic ICD-code), any mental diagnosis (as main diagnosis/cause of hospitalization), and any somatic diagnosis (as main diagnosis/cause of hospitalization) are given in ARFID and controls. As test statistics, incidence rate ratio in ARFID vs. controls (IRR), lower/upper limits of the cluster-robust 95% confidence interval (95% CI LL/UL), cluster-robust standard error (SE), z statistic, raw p-value, and significance according to false discovery rate-adjusted threshold (FDR/Benjamini-Hochberg procedure [BH]) are stated for any diagnosis, any mental diagnosis, and any somatic diagnosis. Statistics were obtained with Poisson regression models (predictor: group [ARFID vs. controls]; covariates: sex [female/male], birth year [1992–2008, factorized]; robust sandwich estimates given clustered twin data; offset term to account for follow-up/exposure time). Significantly different incidence rates in ARFID vs. controls at α_{FDR}=0.0343 are marked by an asterisk (*). *Abbreviation: ARFID, avoidant restrictive food intake disorder.*

Table S8. Sex-stratified Cox regression model estimates in ARFID vs. controls

Condition	Sex: female				Sex: male				Comparison female vs. male				
	HR_{female}	95%CI LL	95%CI UL	p-value	HR_{male}	95%CI LL	95%CI UL	p-value	HR_{female}/HR_{male}	95%CI LL	95%CI UL	p-value	Significant (*) after FDR/BH
E:ShortStature	6.10	3.56	10.44	<0.0001	1.83	0.92	3.62	0.085	3.50	1.47	8.33	0.005	
F:MotorDevelopment	14.10	6.62	30.00	<0.0001	4.90	2.29	10.48	<0.0001	3.16	1.07	9.29	0.037	
Q:DigestiveSystemMalform	4.24	2.30	7.78	<0.0001	1.84	1.10	3.06	0.020	2.38	1.07	5.27	0.033	
Q:ChromosomalAbnorm	12.95	4.89	34.29	<0.0001	6.84	2.71	17.27	<0.0001	1.89	0.49	7.27	0.353	
E:PubertyOnset	3.59	1.40	9.19	0.008	2.20	0.91	5.32	0.081	1.81	0.50	6.62	0.368	
J:ChronicUpperRespiratory	1.77	1.30	2.40	0.0003	1.04	0.78	1.39	0.790	1.72	1.13	2.62	0.011	
ChapterQ:CongenitalConditions	2.52	1.93	3.30	<0.0001	1.61	1.27	2.03	<0.0001	1.60	1.12	2.27	0.009	
J:Rhinitis	2.03	1.31	3.13	0.001	1.30	0.90	1.88	0.170	1.59	0.90	2.82	0.110	
P:PerinatEndocrineMetabolic	1.70	1.06	2.72	0.026	1.13	0.75	1.70	0.569	1.54	0.82	2.87	0.177	
ChapterE:EndocrineConditions	3.44	2.50	4.72	<0.0001	2.30	1.75	3.02	<0.0001	1.52	1.00	2.31	0.051	
Q:CirculatorySystemMalform	2.93	1.89	4.54	<0.0001	1.92	1.18	3.12	0.008	1.51	0.79	2.91	0.213	
EntireChapterC:Cancer	10.50	3.93	28.05	<0.0001	6.67	3.15	14.13	<0.0001	1.51	0.44	5.21	0.515	
AutoimmuneConditions	1.64	1.11	2.41	0.013	1.09	0.74	1.60	0.677	1.51	0.88	2.59	0.138	
F:Autism	12.10	7.59	19.29	<0.0001	8.97	6.66	12.09	<0.0001	1.42	0.81	2.48	0.215	
F:ADHD	5.94	4.05	8.72	<0.0001	4.47	3.46	5.76	<0.0001	1.40	0.88	2.22	0.157	
Q:HeadMalform	2.27	1.06	4.88	0.035	1.87	0.99	3.52	0.053	1.27	0.47	3.44	0.638	
ChapterJ:RespiratoryConditions	1.60	1.33	1.93	<0.0001	1.32	1.13	1.54	0.0004	1.21	0.95	1.55	0.116	
P:PerinatCNSConditions	4.35	1.89	9.99	0.0005	3.83	1.97	7.46	<0.0001	1.20	0.41	3.53	0.734	
K:NoninfectiveIBD	2.88	1.65	5.04	0.0002	2.45	1.49	4.03	0.0004	1.19	0.57	2.48	0.649	
ChapterK:DigestiveConditions	2.24	1.79	2.80	<0.0001	1.92	1.60	2.32	<0.0001	1.18	0.89	1.57	0.260	
K:Constipation	2.41	1.73	3.37	<0.0001	2.09	1.55	2.80	<0.0001	1.16	0.75	1.80	0.501	
F:IntellectualDisability	10.48	6.01	18.26	<0.0001	10.09	7.06	14.42	<0.0001	1.14	0.58	2.23	0.702	
J:AcuteUpperRespiratory	1.57	1.23	2.00	0.0003	1.38	1.13	1.68	0.001	1.14	0.83	1.56	0.409	
K:GERD	7.43	4.17	13.24	<0.0001	6.57	3.96	10.91	<0.0001	1.12	0.52	2.40	0.780	
AllergicConditions	1.68	1.28	2.19	0.0002	1.50	1.23	1.84	<0.0001	1.12	0.80	1.57	0.501	
F:SpeechDevelopment	3.45	1.59	7.47	0.002	3.28	1.99	5.41	<0.0001	1.11	0.44	2.80	0.825	
R:FebrileSeizures	1.20	0.50	2.92	0.684	1.09	0.56	2.11	0.798	1.10	0.36	3.32	0.869	
J:Asthma	1.65	1.20	2.26	0.002	1.53	1.22	1.92	0.0003	1.08	0.73	1.59	0.696	
L:OtherDermatitisEczema	1.52	1.04	2.23	0.032	1.41	1.01	1.99	0.046	1.08	0.65	1.81	0.764	
R:NauseaVomiting	3.36	2.09	5.41	<0.0001	3.16	2.13	4.69	<0.0001	1.07	0.58	1.98	0.836	
P:NeonatalJaundice	1.40	0.99	1.97	0.058	1.32	1.01	1.72	0.039	1.06	0.69	1.64	0.780	

Condition	Sex: female				Sex: male				Comparison female vs. male				
	HR_{female}	95%CI LL	95%CI UL	p-value	HR_{male}	95%CI LL	95%CI UL	p-value	HR_{female}/ HR_{male}	95%CI LL	95%CI UL	p-value	Significant (*) after FDR/BH
P:PerinatRespiratCardiovasc	1.31	0.88	1.95	0.178	1.30	0.96	1.76	0.093	1.04	0.63	1.72	0.873	
K:Hernia	1.34	0.59	3.02	0.487	1.44	1.00	2.06	0.049	1.03	0.42	2.50	0.956	
P:FetalGrowthRetardation	1.25	0.97	1.60	0.081	1.23	1.01	1.51	0.042	1.02	0.74	1.40	0.912	
ChapterF:MentalConditions	3.84	2.96	4.98	<0.0001	3.90	3.25	4.67	<0.0001	1.01	0.73	1.38	0.968	
F:OtherDevelopment	11.38	4.35	29.77	<0.0001	12.37	5.98	25.58	<0.0001	1.01	0.30	3.35	0.988	
P:PerinatInfections	1.80	0.99	3.25	0.053	1.79	1.09	2.94	0.020	1.01	0.48	2.14	0.976	
Q:NervousSystemMalform	11.98	5.87	24.47	<0.0001	10.67	5.42	21.02	<0.0001	0.98	0.36	2.67	0.970	
ChapterP:PerinatalConditions	1.20	0.95	1.51	0.120	1.25	1.05	1.50	0.014	0.97	0.72	1.29	0.813	
J:AcuteLowerRespiratory	1.55	1.12	2.13	0.008	1.62	1.26	2.08	0.0001	0.95	0.63	1.42	0.786	
Q:MusculoskeletalMalform	1.95	1.19	3.20	0.009	2.05	1.39	3.04	0.0003	0.95	0.51	1.80	0.887	
K:OtherUpperGastrointest	4.81	2.35	9.81	<0.0001	5.23	2.75	9.95	<0.0001	0.87	0.33	2.28	0.776	
P:MaternalComplications	1.03	0.64	1.67	0.903	1.19	0.83	1.71	0.338	0.86	0.47	1.58	0.630	
P:PerinatFeeding	1.86	0.93	3.73	0.079	2.21	1.20	4.06	0.011	0.85	0.34	2.13	0.725	
E:VolumeDepletion	2.44	1.10	5.42	0.029	2.98	1.78	4.99	<0.0001	0.81	0.31	2.10	0.666	
P:PerinatHematologic	1.11	0.53	2.33	0.783	1.46	0.83	2.59	0.192	0.76	0.30	1.93	0.562	
S:IntracranialInjury	0.94	0.53	1.67	0.844	1.29	0.89	1.88	0.175	0.73	0.37	1.44	0.366	
L:Urticaria	1.15	0.57	2.32	0.687	1.62	1.00	2.62	0.049	0.71	0.30	1.66	0.432	
K:Stomatitis	2.29	0.95	5.52	0.064	3.44	1.86	6.34	<0.0001	0.67	0.24	1.82	0.427	
ChapterI:CirculatoryConditions	1.44	0.64	3.25	0.381	2.21	1.36	3.60	0.001	0.65	0.25	1.70	0.381	
K:CeliacDisease	1.71	0.81	3.63	0.161	2.62	1.34	5.13	0.005	0.64	0.23	1.76	0.383	
G:CerebralPalsy	4.42	2.06	9.50	0.0001	7.27	4.36	12.12	<0.0001	0.59	0.23	1.47	0.257	
G:Epilepsy	3.42	1.67	7.00	0.0008	7.48	4.99	11.23	<0.0001	0.46	0.20	1.06	0.069	
R:Dysphagia	6.18	2.66	14.35	<0.0001	14.21	8.45	23.89	<0.0001	0.43	0.16	1.15	0.093	
F:OtherAnxietyConditions	1.46	0.69	3.09	0.325	3.70	2.11	6.50	<0.0001	0.38	0.15	0.97	0.043	

Sex-specific hazard ratios in female individuals with ARFID vs. female controls (HR_{female}) and male individuals with ARFID vs. male controls (HR_{male}), lower/upper limits of the cluster-robust 95% confidence interval (95% CI LL/UL), and raw p-values are stated for each analyzed condition (conditions with n<5 individuals per sex in ARFID or controls were not analyzed) from the sex-stratified Cox regression model (predictor: group [ARFID vs. controls]; sex strata [female/male strata]; covariate: birth year [1992–2008, factorized]; robust sandwich estimates given clustered twin data). Sex-specific HRs were compared (HR_{female}/HR_{male}) and statistics of the comparison are stated: lower/upper limits of the cluster-robust 95% confidence interval of HR_{female}/HR_{male}, raw p-value, and significance according to the false discovery rate-adjusted threshold (FDR/Benjamini-Hochberg procedure [BH]). There were no statistically significant sex differences (HR_{female} vs. HR_{male}) in ARFID vs. controls at α_{FDR}=0.00014 (across 103 supplementary tests). Nominally significant sex differences (p<0.050) are highlighted with light-orange background. Conditions are sorted in descending order of HR_{female}/HR_{male} for easier orientation. *Abbreviations: ADHD, attention deficit hyperactivity disorder; ARFID, avoidant restrictive food intake disorder; CNS, central nervous system; GERD, gastro-esophageal reflux disorder; IBD, inflammatory bowel disease.*

Table S9. Sex effects on number of distinct overall, mental, and somatic diagnoses in ARFID vs. controls

Condition	ARFID				Controls				Poisson regression model statistics						
	Female		Male		Female		Male		Group-*x*-sex interaction effect						
	Mean	SEM	Mean	SEM	Mean	SEM	Mean	SEM	IRR[a]	95%CI LL	95%CI UL	Robust SE	z statistic	p-value	Significant (*) after FDR/BH
All diagnoses	5.85	0.44	6.53	0.37	2.54	0.03	3.01	0.03	1.05	0.87	1.27	0.10	0.55	0.580	
Mental diagnoses	0.77	0.10	1.16	0.10	0.19	0.01	0.25	0.01	0.85	0.61	1.18	0.17	-0.96	0.335	
Somatic diagnoses	5.08	0.41	5.38	0.32	2.36	0.02	2.77	0.03	1.11	0.90	1.35	0.10	0.97	0.330	

Mean and standard error of the mean (SEM) for number of *all* distinct diagnoses (i.e., unique diagnostic ICD-codes), number of distinct *mental* diagnoses, and number of distinct *somatic* diagnoses per individual twin are given in female and male individuals with ARFID and female and male controls. As test statistics, incidence rate ratio of the group (ARFID vs. controls)-*x*-sex interaction effect (IRR), lower/upper limits of the cluster-robust 95% confidence interval (95% CI LL/UL), cluster-robust standard error (SE), z statistic, raw p-value, and significance according to supplementary false discovery rate-adjusted threshold (FDR/Benjamini-Hochberg procedure [BH]) are stated for all distinct diagnoses, distinct mental diagnoses, and distinct somatic diagnoses. Statistics were obtained with Poisson regression models including a group-*x*-sex interaction term (other predictors/main effects: group [ARFID vs. controls], sex [female/male]; covariate: birth year [1992–2008, factorized]; robust sandwich estimates given clustered twin data; offset term to account for follow-up/exposure time). There were no significant group-*x*-sex interaction effects at α_{FDR}=0.00014 (across 103 supplementary tests). [a]IRRs>1 indicate greater incidence rates in females with ARFID than males with ARFID. IRRs<1 indicate greater incidence rates in males with ARFID than females with ARFID (regarding directionality [*not* statistical significance]). *Abbreviation: ARFID, avoidant restrictive food intake disorder.*

Table S10. Sex effects on number of inpatient days due to any, any mental, and any somatic diagnosis in ARFID vs. controls

Condition	ARFID				Controls				Poisson regression model statistics						
	Female		Male		Female		Male		Group-*x*-sex interaction effect						
	Mean	SEM	Mean	SEM	Mean	SEM	Mean	SEM	IRR[a]	95%CI LL	95%CI UL	Robust SE	z statistic	p-value	Significant (*) after FDR/BH
Any diagnosis	29.57	5.61	24.71	2.65	8.82	0.19	9.54	0.19	1.28	0.82	1.97	0.22	1.09	0.274	
Mental diagnosis	2.05	1.48	0.32	0.12	0.39	0.07	0.09	0.02	1.35	0.25	7.32	0.86	0.34	0.730	
Somatic diagnosis	25.44	5.07	21.48	2.34	8.00	0.16	8.96	0.18	1.31	0.83	2.06	0.23	1.17	0.244	

Mean and standard error of the mean (SEM) for number of inpatient days per individual twin due to any diagnosis (i.e., unique diagnostic ICD-code), any mental diagnosis (as main diagnosis/cause of hospitalization), and any somatic diagnosis (as main diagnosis/cause of hospitalization) are given in female and male individuals with ARFID and female and male controls. As test statistics, incidence rate ratio of the group (ARFID vs. controls)-*x*-sex interaction effect (IRR), lower/upper limits of the cluster-robust 95% confidence interval (95% CI LL/UL), cluster-robust standard error (SE), z statistic, raw p-value, and significance according to supplementary false discovery rate-adjusted threshold (FDR/Benjamini-Hochberg procedure [BH]) are stated for any diagnosis, any mental diagnosis, and any somatic diagnosis. Statistics were obtained with Poisson regression models including a group-*x*-sex interaction term (other predictors/main effects: group [ARFID vs. controls], sex [female/male]; covariate: birth year [1992–2008, factorized]; robust sandwich estimates given clustered twin data; offset term to account for follow-up/exposure time). There were no significant group-*x*-sex interaction effects at α_{FDR}=0.00014 (across 103 supplementary tests). [a]IRRs>1 indicate greater incidence rates in females with ARFID than males with ARFID. IRRs<1 indicate greater incidence rates in males with ARFID than females with ARFID (regarding directionality [*not* statistical significance]). *Abbreviation: ARFID, avoidant restrictive food intake disorder.*

Table S11. Age group-stratified Cox regression model estimates in ARFID vs. controls

Condition	Age group 1: 0 to <6 years				Age group 2: 6 to <12 years				Age group 3: 12 to <18 years				Comparison age groups 1 vs. 2					Comparison age groups 3 vs. 2				
	$HR_{age\ group\ 1}$	95%CI LL	95%CI UL	p-value	$HR_{age\ group\ 2}$	95%CI LL	95%CI UL	p-value	$HR_{age\ group\ 3}$	95%CI LL	95%CI UL	p-value	$HR_{age\ group\ 1}$/ $HR_{age\ group\ 2}$	95%CI LL	95%CI UL	p-value	Sig. (*) after FDR	$HR_{age\ group\ 3}$/ $HR_{age\ group\ 2}$	95%CI LL	95%CI UL	p-value	Sig. (*) after FDR
F:Autism	16.09	9.40	27.56	<0.0001	12.35	8.75	17.43	<0.0001	4.32	2.54	7.34	<0.0001	1.30	0.69	2.46	0.413		0.35	0.19	0.66	0.001	
ChapterF:MentalConditions	5.17	3.95	6.77	<0.0001	4.48	3.67	5.47	<0.0001	2.12	1.52	2.95	<0.0001	1.15	0.83	1.61	0.399		0.47	0.32	0.70	0.0001	*
ChapterI:CirculatoryConditions	2.19	1.12	4.30	0.023	2.50	1.36	4.59	0.003	1.17	0.48	2.83	0.733	0.88	0.35	2.18	0.777		0.47	0.17	1.28	0.140	
ChapterE:EndocrineConditions	3.10	2.32	4.15	<0.0001	2.97	2.10	4.21	<0.0001	1.53	0.89	2.65	0.126	1.04	0.66	1.64	0.858		0.52	0.27	0.98	0.044	
F:ADHD	9.14	4.33	19.32	<0.0001	5.87	4.51	7.65	<0.0001	3.10	2.08	4.60	<0.0001	1.56	0.70	3.44	0.274		0.53	0.33	0.85	0.009	
Q:MusculoskeletalMalform	1.70	1.12	2.57	0.013	3.37	1.92	5.93	<0.0001	1.79	0.79	4.04	0.16	0.50	0.25	1.01	0.055		0.53	0.20	1.43	0.211	
F:IntellectualDisability	16.18	9.62	27.21	<0.0001	9.82	6.29	15.33	<0.0001	5.98	2.82	12.66	<0.0001	1.65	0.83	3.26	0.152		0.61	0.25	1.46	0.265	
S:IntracranialInjury	1.45	0.94	2.25	0.092	1.12	0.66	1.91	0.667	0.74	0.33	1.65	0.461	1.29	0.65	2.57	0.461		0.66	0.25	1.73	0.394	
K:NoninfectiveIBD	2.02	1.21	3.38	0.008	4.42	2.18	8.97	<0.0001	2.96	1.19	7.39	0.02	0.46	0.19	1.10	0.081		0.67	0.21	2.12	0.495	
J:Asthma	1.54	1.23	1.92	0.0001	1.77	1.22	2.57	0.002	1.22	0.61	2.44	0.584	0.87	0.57	1.33	0.514		0.69	0.31	1.51	0.350	
ChapterK:DigestiveConditions	1.95	1.61	2.35	<0.0001	2.33	1.79	3.03	<0.0001	1.77	1.19	2.64	0.005	0.84	0.61	1.15	0.267		0.76	0.47	1.23	0.263	
J:ChronicUpperRespiratory	1.41	1.08	1.85	0.013	1.21	0.83	1.78	0.322	0.97	0.51	1.87	0.937	1.16	0.74	1.84	0.516		0.80	0.38	1.72	0.572	
ChapterJ:RespiratoryConditions	1.43	1.25	1.63	<0.0001	1.39	1.04	1.85	0.028	1.30	0.84	2.02	0.238	1.03	0.75	1.42	0.847		0.94	0.55	1.59	0.817	
J:AcuteUpperRespiratory	1.38	1.16	1.64	0.0002	1.80	1.21	2.69	0.004	1.75	0.93	3.26	0.081	0.76	0.50	1.18	0.225		0.97	0.46	2.03	0.930	
J:Rhinitis	1.25	0.62	2.54	0.53	1.57	1.08	2.29	0.017	1.56	0.91	2.68	0.107	0.80	0.36	1.77	0.577		0.99	0.51	1.91	0.979	
K:Constipation	1.93	1.39	2.69	<0.0001	2.50	1.75	3.56	<0.0001	2.56	1.36	4.85	0.004	0.77	0.47	1.26	0.303		1.03	0.50	2.13	0.944	
AllergicConditions	1.52	1.24	1.87	<0.0001	1.59	1.17	2.15	0.003	1.65	1.06	2.57	0.027	0.96	0.67	1.38	0.815		1.04	0.61	1.76	0.892	
ChapterQ:CongenitalConditions	1.96	1.60	2.40	<0.0001	1.43	0.88	2.32	0.154	2.15	1.24	3.72	0.006	1.38	0.81	2.33	0.234		1.51	0.72	3.14	0.274	
R:Dysphagia	11.84	6.34	22.12	<0.0001	7.25	3.27	16.08	<0.0001	13.86	5.85	32.81	<0.0001	1.63	0.59	4.49	0.342		1.91	0.59	6.16	0.278	
AutoimmuneConditions	1.36	0.95	1.95	0.093	0.90	0.51	1.59	0.717	1.77	0.99	3.15	0.053	1.51	0.78	2.95	0.223		1.97	0.87	4.43	0.103	
L:OtherDermatitisEczema	1.53	1.10	2.12	0.012	0.86	0.49	1.53	0.616	2.45	1.47	4.10	0.0006	1.77	0.91	3.43	0.093		2.84	1.32	6.11	0.008	

Age group-specific hazard ratios in 0 to <6-year-old individuals with ARFID vs. controls of the same age group ($HR_{age\ group\ 1}$), 6 to <12-year-old individuals with ARFID vs. controls ($HR_{age\ group\ 2}$), and 12 to <18-year-old individuals with ARFID vs. controls ($HR_{age\ group\ 3}$), lower/upper limits of the cluster-robust 95% confidence interval (95% CI LL/UL), and p-values are stated for each analyzed condition (conditions with n<5 individuals per age group in ARFID or controls were not analyzed) from the age group-stratified Cox regression model (predictor: group [ARFID vs. controls]; age/time group strata [age groups 1-3]; covariates: sex [female/male], birth year [1992–2008, factorized]; robust sandwich estimates given clustered twin data). Age group-specific HRs were compared ($HR_{age\ group\ 1}/HR_{age\ group\ 2}$ and $HR_{age\ group\ 3}/HR_{age\ group\ 2}$) and statistics of both comparisons are stated: lower/upper limits of the cluster-robust 95% confidence interval of $HR_{age\ group\ 1}/HR_{age\ group\ 2}$ and $HR_{age\ group\ 3}/HR_{age\ group\ 2}$, raw p-value, and significance according to the false discovery rate-adjusted threshold (FDR/Benjamini-Hochberg procedure [BH]). There was one significant age group difference ($HR_{age\ group\ 3}$ vs. $HR_{age\ group\ 2}$ for ChapterF:MentalConditions, marked by an asterisk [*] and orange background) in ARFID vs. controls at α_{FDR}=0.00014 (across 103 supplementary tests). All other nominally significant age group differences (p<0.050) are highlighted with light-orange background. Conditions are sorted in ascending order of $HR_{age\ group\ 3}/HR_{age\ group\ 2}$ for easier orientation. *Abbreviations: ADHD, attention deficit hyperactivity disorder; ARFID, avoidant restrictive food intake disorder; IBD, inflammatory bowel disease.*

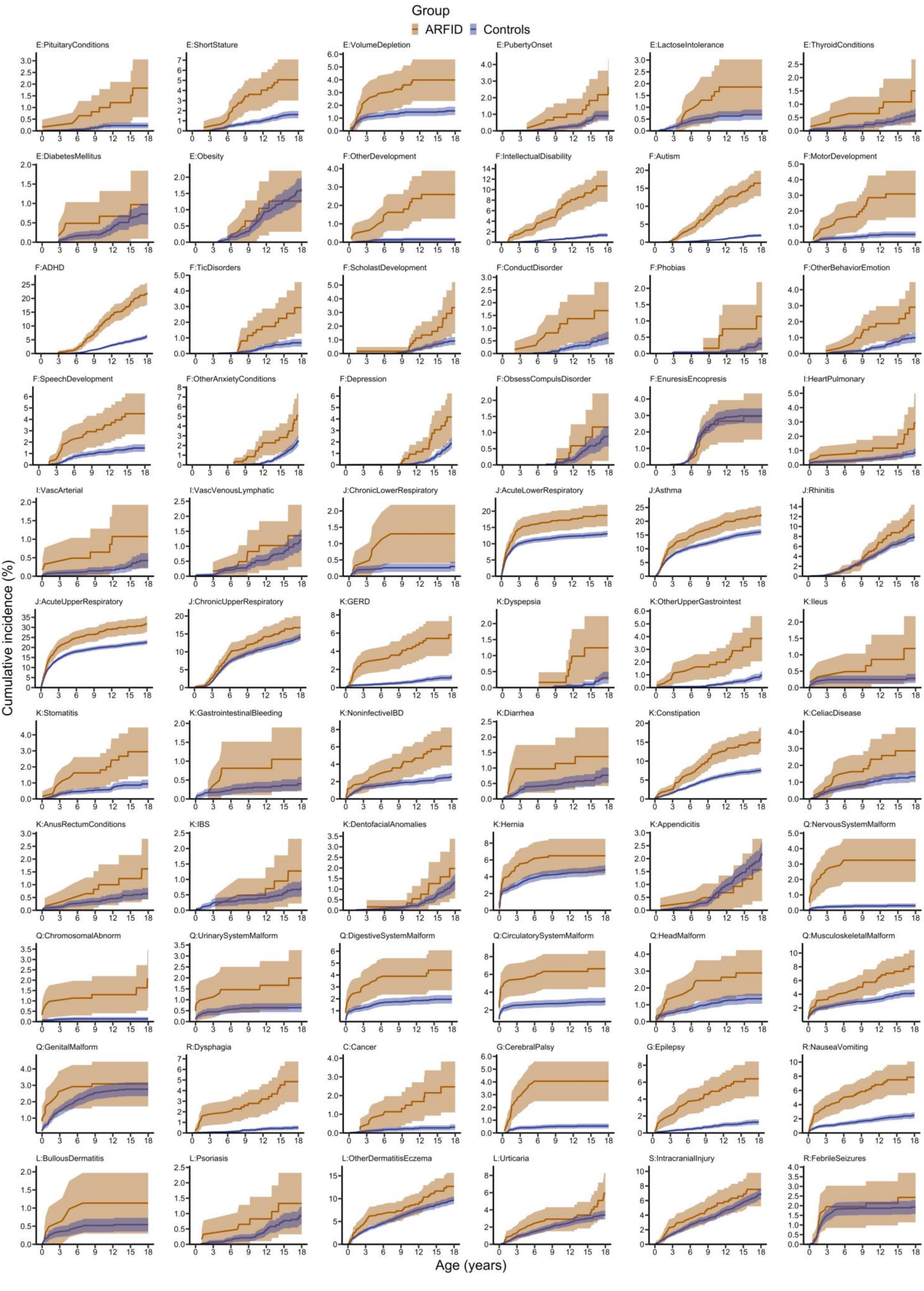
Group
ARFID
Controls
E:PituitaryConditions
E:ShortStature
E:VolumeDepletion
E:PubertyOnset
E:LactoseIntolerance
E:ThyroidConditions
E:DiabetesMellitus
E:Obesity
F:OtherDevelopment
F:IntellectualDisability
F:Autism
F:MotorDevelopment
F:ADHD
F:TicDisorders
F:ScholastDevelopment
F:ConductDisorder
F:Phobias
F:OtherBehaviorEmotion
F:SpeechDevelopment
F:OtherAnxietyConditions
F:Depression
F:ObsessCompulsDisorder
F:EnuresisEncopresis
I:HeartPulmonary
I:VascArterial
I:VascVenousLymphatic
J:ChronicLowerRespiratory
J:AcuteLowerRespiratory
J:Asthma
J:Rhinitis
J:AcuteUpperRespiratory
J:ChronicUpperRespiratory
K:GERD
K:Dyspepsia
K:OtherUpperGastrointest
K:Ileus
K:Stomatitis
K:GastrointestinalBleeding
K:NoninfectiveIBD
K:Diarrhea
K:Constipation
K:CeliacDisease
K:AnusRectumConditions
K:IBS
K:DentofacialAnomalies
K:Hernia
K:Appendicitis
Q:NervousSystemMalform
Q:ChromosomalAbnorm
Q:UrinarySystemMalform
Q:DigestiveSystemMalform
Q:CirculatorySystemMalform
Q:HeadMalform
Q:MusculoskeletalMalform
Q:GenitalMalform
R:Dysphagia
C:Cancer
G:CerebralPalsy
G:Epilepsy
R:NauseaVomiting
L:BullousDermatitis
L:Psoriasis
L:OtherDermatitisEczema
L:Urticaria
S:IntracranialInjury
R:FebrileSeizures
Cumulative incidence (%)
Age (years)

Figure S1. Cumulative incidence plots in ARFID vs. controls for individual and grouped conditions

Cumulative incidence of having a *specific* condition (%) and lower and upper limits of its cluster-robust 95% confidence interval are plotted over age (0 to <18 years) in ARFID vs. controls. The Kaplan-Meier-based estimation method is explained in the statistical analysis section of the main article and in the legend of Table S5. Conditions are sorted in accordance with Table S4, i.e., in descending order of base Cox regression model hazard ratios within ICD-chapters, the group of further conditions, and grouped allergic and autoimmune conditions (please consult Figure 4 in the main article for cumulative incidence plots for ICD-*chapters* and grouped allergic and autoimmune conditions; perinatal conditions excluded given their occurrence during the perinatal period and lack of age-related dynamics). *Abbreviations: ADHD, attention deficit hyperactivity disorder; ARFID, avoidant restrictive food intake disorder; GERD, gastro-esophageal reflux disorder; IBD, inflammatory bowel disease; IBS, irritable bowel syndrome.*

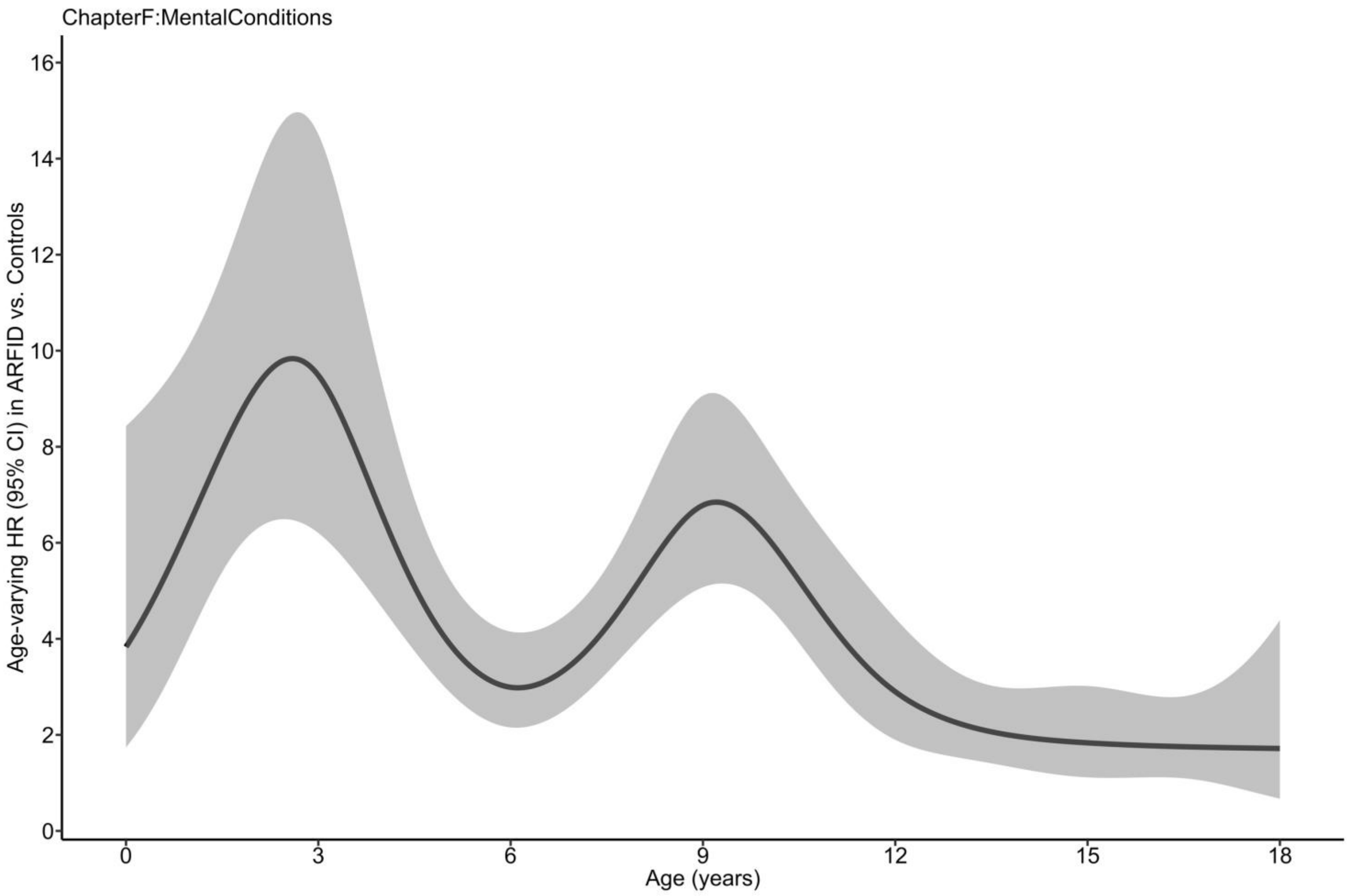

Figure S2. Age-varying hazard ratios in ARFID vs. controls for ICD-chapter F: mental conditions

Following-up on significant age group differences ($HR_{age\ group\ 3}$ vs. $HR_{age\ group\ 2}$) in ARFID vs. controls for ICD-chapter F: mental conditions (Table S11), time-varying hazard ratios (HRs) in ARFID vs. controls and lower and upper limits of their cluster-robust 95% confidence intervals are plotted over age (0 to <18 years). The curve was estimated using the base Cox regression model (predictor: group [ARFID vs. controls]; covariates: sex [female/male], birth year [1992–2008, factorized]; robust sandwich estimates given clustered twin data) and adding age-/time-related variance via cubic splines with pre-specified knots at 3, 6, 9, 12, and 15 years of age (boundary knots at 0 and <18 [≈17.9] years of age). *Abbreviation: ARFID, avoidant restrictive food intake disorder.*
